# Systematic review of worldwide variations of the prevalence of wheezing symptoms in children

**DOI:** 10.1186/1476-069X-7-57

**Published:** 2008-11-10

**Authors:** Swatee P Patel, Marjo-Riitta Järvelin, Mark P Little

**Affiliations:** 1 School of Health and Social Care, the University of Greenwich, Southwood Site, Avery Hill Road, London SE9 2UG, UK; 2 Department of Epidemiology and Public Health, Imperial College Faculty of Medicine, Norfolk Place, London W2 1PG, UK

## Abstract

**Background:**

Considerable variation in the prevalence of childhood asthma and its symptoms (wheezing) has been observed in previous studies and there is evidence that the prevalence has been increasing over time.

**Methods:**

We have systematically reviewed the reported prevalence and time trends of wheezing symptoms among children, worldwide and within the same country over time. All studies comprising more than 1000 persons and meeting certain other quality criteria published over a 16-year period, between January 1990 and December 2005, are reported and a comparison of ISAAC (International Study of Asthma and Allergies in Childhood) and non-ISAAC studies is made, in part as a way of expanding the power to examine time trends (the older studies tend to be non-ISAAC), but also to examine possible methodological differences between ISAAC and non-ISAAC questions.

**Results:**

A wide range of current prevalence of wheeze was observed between and within countries over time. The UK had the highest recorded prevalence of 32.2% in children aged 13–14 in 1994–5 and Ethiopia had the lowest prevalence, 1.7% in children aged 10–19 in 1996. All studies in Australia and the UK were compared using multiple logistic regression. ISAAC phase I and III studies reported significantly higher prevalence of current wheeze (OR = 1.638) compared with non-ISAAC studies, after adjusting for various other factors (country, survey year, age of child, parental vs child response to the survey). Australia showed a significantly higher prevalence of current wheezing (OR = 1.343) compared with the UK, there was a significant increase in the prevalence odds ratio per survey year (2.5% per year), a significant decrease per age of child (0.7% per year), and a significantly higher response in current wheezing if the response was self-completed by the child (OR = 1.290). These factors, when explored separately for ISAAC and non-ISAAC studies, showed very different results. In ISAAC studies, or non-ISAAC studies using ISAAC questions, there was a significant decrease in current wheezing prevalence over time (2.5% per year). In non-ISAAC studies, which tend to cover an earlier period, there was a significant increase (2.6% per year) in current wheezing prevalence over time. This is very likely to be a result of prevalence of wheezing increasing from the 1970s up to the early 1990s, but decreasing since then.

**Conclusion:**

The UK has the highest recorded prevalence of wheezing and Ethiopia the lowest. Prevalence of wheezing in Australia and the UK has increased from the 1970s up to the early 1990s, but decreased since then and ISAAC studies report significantly higher prevalences than non-ISAAC studies.

## Background

Considerable variation in the prevalence of childhood asthma and its symptoms (in particular, wheezing) has been observed in previous studies and there is evidence that the prevalence has been increasing over time. These differences may, in part, be due to geographical variations and due to methodological problems in defining asthma symptoms.

There is a multiplicity of endpoints used to define and diagnose asthma in an individual. For example, diagnosis is often based on a detailed medical history, including family health history, combined with examination of the upper and lower respiratory tract [1]. Typically, this information is combined with information from laboratory tests. However, diagnostic criteria often differ between doctors in the same locality as well as between countries, and access to health care in different countries can also have an influence on the reported prevalence of doctor-diagnosed asthma.

Epidemiological studies have used different methods of measuring asthma prevalence and its symptoms in surveys. Questionnaires are administered, and depending on the wording of the questions asked, there has been variation in the symptoms elicited. The symptoms may not be present on a particular day, so a one-year period prevalence is often used in epidemiological studies to allow for seasonal variation.

In self-reported asthma, questions are usually asked about wheezing, chest tightness, breathlessness and coughing, but studies have shown that wheezing is the most important symptom for the identification of asthma in epidemiological studies [1,2]. Some studies have shown that self-reported wheeze has reasonably good specificity and sensitivity for bronchial hyper-responsiveness both in children and adults [3-5]. Wheeze is rarely a symptom of other diseases, in particular emphysema or chronic bronchitis, which are rare in children, but it is very often indicative of acute viral infection, which is common in this age group [6].

Doctor diagnosed asthma has been shown to have a lower prevalence than the actual symptoms reported by the individual [1,7-9]. Until the early 1990's, there was no standardized method of comparing asthma prevalence between countries. Only a small number of studies had used common protocols [9-13]. In 1991 the International Study of Asthma and Allergies in Childhood (ISAAC) was set up to achieve uniform diagnostic criteria [14]. Their first worldwide epidemiological study, Phase I, was carried out in 1994–95. It included 56 countries and reported the prevalence of asthma symptoms in 6–7 year old children and in 13–14 year old adolescents [15]. The Phase III study used the same research design as Phase I, but was carried out in 2002–03 [16]. The Phase II study comprised a much more detailed investigation of possible correlates of childhood asthma, in particular eczema, and in contrast to ISAAC Phase I and III used 9–11 year old children [17,18]. The ISAAC questionnaire is now widely used to assess self-diagnosed asthma by asking about the symptoms [19].

This review has been carried out to assess and summarise the extent of the literature published on wheezing symptoms in children, which includes not only ISAAC but also all non-ISAAC studies that fulfilled specific quality criteria. There are many studies published which are not ISAAC and it is worthwhile to combine the published literature in a review such as this. We report the prevalence and time trends of current symptoms of childhood wheezing in the past 12 months in all studies, worldwide and within the same region over different time periods, and compare the results of ISAAC and non-ISAAC studies. A particular focus of parts of the analysis are studies in the UK and Australia, because of the large number of studies carried out in these two countries – we examine in some detail differences in time trends of wheeze between the two countries. As we shall see, there are distinct, and perhaps surprising, differences between these two developed countries. In what follows one should note the distinction between the underlying medical condition, "asthma" and its principal symptom, "wheezing"; however, as above, we are referring in all cases to studies of wheezing symptoms.

## Methods

Studies included in this systematic review had to satisfy the following requirements:

1. listed in Medline or Embase databases;

2. published in the period January 1990 to December 2005;

3. using the keywords: 'prevalence' AND

'asthma OR wheeze OR wheezing' AND

'child OR children OR adolescent';

4. full journal articles (rather than abstracts) published in English;

5. epidemiological studies of sample size greater than 1000;

6. prevalence of 'current wheezing' is reported.

In most epidemiological studies of the prevalence of asthma symptoms, two main types of questions are used.

(i) 'Current' asthma/wheezing, which is normally a period prevalence, and where the question asked is often of the form "Have you had asthma/wheezing *in the past 12 months*?"

(ii) 'Lifetime' asthma/wheezing, in which the question is often "Have you *ever *had asthma/wheezing at anytime in the past?"

Estimates of current prevalence are likely to be more reliable, although Kuehni *et al *have shown that retrospective recall of wheeze at age 8–13 years is a valid proxy measure for lifetime prevalence of wheeze [20]. Questions which are similar to: "In the past 12 months, has the child had wheezing/whistling in the chest?" (from ISAAC questionnaire), i.e. current wheezing, will be used in this review (see footnote '*Prevalence*' in Additional File 1). Examples of questions that were asked, and could not be included in this review were: doctor diagnosed asthma (as asthma diagnosis varies by countries); self-reported asthma or asthma attacks in the last 12 months; wheeze ever or occasional wheeze ever (for which there could be recall problems).

A minimum sample size of 1000 was used, as recommended by the ISAAC Steering Committee for small populations [15], to obtain good estimates of wheezing prevalence.

For each selected article in this review, the following information was extracted and given in Additional Files 1, 2, 3, 4, 5:

*Country *Country in which the study was carried out

*Reference *Reference number of the journal article

*Survey year *Year in which the survey was carried out, if reported, otherwise the year prior to publication year

*Area *City/region

*N (Response rate) *Number of questionnaires returned and response rate

*Age (years) *Age range of children sampled (years)

*Ascertainment *Method of ascertainment of wheeze (P = Parental-report, S = Self-report)

*Prevalence % *The percentage of children (boys and girls combined) who responded 'Yes' to the particular wheezing question (see footnote in Additional File 1 for different types of questions) out of the total number of children who answered that question, and type of question asked

*95% CI *95% confidence interval for the prevalence (if unreported in the article then CI for a single proportion was calculated based on the prevalence and sample size)

In the ISAAC questionnaire, limited agreement has been shown between the written and video questionnaires of symptoms of asthma; the video questionnaire giving lower prevalence rates in 13–14 year old adolescents in two Canadian communities [21]. In the studies reported here, if both methods are used then the written questionnaire results were reported.

To investigate time trends within Australia and the UK, multiple logistic regression models were fitted using wheezing prevalence as the outcome variable. The standard log-linear logistic model was used, so that the probability of an individual in stratum *i *being affected by wheeze was assumed to be:

(1) $$P[\text{individual in stratum }i\text{ has wheeze}]=\frac{\exp[\alpha_{0}+\sum_{i}\alpha_{i}z_{ji}]}{1+\exp[\alpha_{0}+\sum_{i}\alpha_{i}z_{ji}]}$$

and where (*z*_*ji*_)_*j *_are the set of variables (country, year, ISAAC vs non-ISAAC study etc) associated with that stratum. The models were fitted via binomial maximum likelihood using SPSS and the odds ratios, that is to say the quantities, exp [*α*_*i*_], and 95% profile-likelihood confidence intervals are reported [22]. For studies where the year of survey was not reported in the article for Australia and the UK, the year prior to the year of publication was used as survey year in the logistic regression analysis.

## Results

From the literature search, 2,839 abstracts were listed in Medline, and 2,844 in Embase, from which 712 full articles were selected for further investigation after reviewing the abstracts. From these, 180 satisfied the above criteria. Some articles had referred to the same data set, thus there were 127 distinct studies reported in this review.

### Prevalence

Additional Files 1, 2, 3, 4, 5 give the prevalence of current wheezing in children for the five continents. There is a very wide range of current prevalence of wheeze between and within different countries. The UK reported the highest prevalence of current wheeze in the world, 32.2%, in children aged 13–14 in 1994–1995 [15]. Ethiopia had the lowest prevalence, 1.7% in children aged 10–19 in 1996 [23].

### Studies in North and South America

Additional File 1 shows the studies carried out in North and South America; among these countries the USA had the largest number of published studies. A nationwide survey in the USA between 1988–1994 showed that the current prevalence of wheezing amongst 2–3 year olds was 26.4% and amongst 9–11 year olds was 13.4% [24]. The highest prevalence rates were recorded in North Carolina, 26.1% in children aged 13–14 in 1999–2000 [25].

In the rest of North America, Canada had recorded substantially higher prevalence rates in children aged 13–14 (30.6% in Hamilton and 24.0% in Saskatoon) than in children aged 6–7 (20.1% in Hamilton and 14.1% in Saskatoon) [15]. The study in Montreal [26] in 6–12 year olds showed very low prevalence of current wheezing, 5.1%; it has been shown that this is likely to be due to unsatisfactory translation of the term wheezing into French, in another study carried out in Quebec [27]. Mexico had the lowest prevalence of current wheezing (< 10%) [15,28]. In Central America, both Costa Rica and Panama showed very high prevalence of wheezing (32.1% and 23.5% respectively) in 6–7 year old children in 1995 [15].

In studies carried out in South America high prevalence rates were observed in Chile [12] (17.2% in 15-yr-olds to 26.2% in 7-yr-olds), as early as 1990. In Brazil, the ISAAC Phase I study [15] carried out in 1994–95 and the same ISAAC questionnaire methodology used in a study amongst 6–7 and 13–14 year olds carried out in 1999 in one of the same centres as the Phase I study [29], both showed higher prevalence of current wheezing than in the non-ISAAC study carried out in 1994, using the same ISAAC methodology in two non-ISAAC centres (iron-mining cities in a mountainous region).

### Studies in Europe

Of the five continental groups Europe had the largest number of published studies overall (Additional File 2). The UK had the highest prevalence, of 32.2% in 1994–5, in the ISAAC Phase I study of 35,485 adolescents [15,30]. Low prevalence rates, of less than 10%, were observed in Albania, Austria, Belgium, Cyprus, Estonia, Finland, France, Georgia, Greece, Hungary, Italy, Latvia, Malta, Romania, the Slovak Republic, and Switzerland in children aged 6–10, whereas Bulgaria (14.5%) [31], the Czech Republic (14.7%) [31], Ireland (17.4%) [32], and Norway (13.6%) [33] had markedly higher prevalence rates.

In the UK, national studies of the prevalence of asthma symptoms (wheezing) reported in 1986 that 6.6% of 16-year-olds had wheezing in the past year [34] and by 1995 this had increased to 32.3% among 12–14 year old children [15], using comparable questions. In the younger age group (6–10 years) in the UK, the current prevalence of wheezing ranged from 7.6% in 1980 [34] to 20.2% in 1999 [35], using comparable questions.

### Studies in the Eastern Mediterranean and Africa

Apart from the ISAAC studies conducted in 1994–95, very few countries had carried out epidemiological studies of asthma in the Eastern Mediterranean and Africa, reported in English (Additional File 3).

In Africa, very low prevalence rates were observed in Ethiopian rural communities (2.0% in 0–9 year olds, 1.7% in 10–19 year olds) [23], intermediate levels of wheeze prevalence (5%–14%) were observed in Algeria, Kenya, Morocco and Nigeria and the highest rates were in South Africa, 26.8% in 7–8 year olds in 1993 [36].

In the Eastern Mediterranean, Iran, Oman and Palestine (West Bank) had the lowest prevalence of wheeze (< 11%), while the highest rates were observed in Israel (17.9%) [37], Kuwait (16.1%) [38], and Malta (16.0%) [15], amongst 13–14 year old adolescents. In Turkey, many of the studies had not used the ISAAC question and the prevalence of wheeze was low.

### Studies in Asia

Amongst studies conducted in Asia, low prevalence rates (< 9%) were observed in China, Hong Kong, India, Indonesia and Malaysia while Japan (17.3%) [15], Korea (13.6%) [39] and Singapore (15.7% in 1994 and 10.2% in 2001) [15,40] had higher prevalence rates in 6–7 year olds (Additional File 4). The majority of the studies reported had used the ISAAC questions relating to current wheeze.

### Studies in Australasia

Australia, New Zealand and Fiji had a very high prevalence of current wheezing with the majority of the studies showing the prevalence of current wheezing in the range 18% – 30% (Additional File 5). The highest prevalence of 30.2% was observed in New Zealand [15] amongst a very large sample of 13–14 year olds in 1992–93, followed by Australia [9] which observed a prevalence of 29.7% amongst 12–15 year olds in 1991. Fiji [41] reported a prevalence of 21.0% in 1990 amongst 9–10 year old children.

### Subgroup analysis: Studies in Australia and the UK

Australia and the UK had the largest number of studies carried out and published (14 and 25 publications respectively over the 16-year period), and these are investigated further to assess differences in prevalence and trends in prevalence between the two countries.

**Figure 1:**
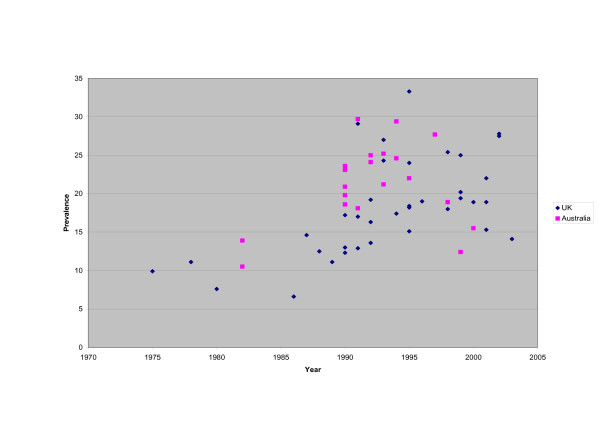
Prevalence of "wheezing in the last year" by calendar year of survey in all children (aged 0–16), reported in published studies in Australia and UK.

The overall trend of the current wheeze prevalence, by calendar year for both Australia and UK can be seen in Figure 1. This shows overall (over the period 1990–2005) an increasing trend for both countries. Table 1 shows the findings of multiple logistic regression analysis of the effects of country (UK versus Australia), year of survey, age of child, parental- or self-report questionnaire, and if the study was an ISAAC study or not, on the current prevalence of wheeze. This allows each factor to be assessed while adjusting for the other factors. When using the data from all studies, Australia showed a statistically significant increase in prevalence compared with the UK, OR = 1.343 [95% CI 1.318, 1.369] allowing for all other factors. There was also a statistically significant increase in the prevalence per survey year (2.5% per year; 95% CI 2.3%, 2.6%), and a significant decrease per year of age of child (0.7% per year; 95% CI 0.3%, 1.1%). If the study was an ISAAC study, phase I or III, then the odds of wheezing was significantly higher compared with non-ISAAC studies [OR = 1.638; 95% CI 1.598, 1.678], after adjusting for the other factors. Similar results were obtained when comparing ISAAC question with a non-ISAAC question (OR = 1.331; 95% CI 1.304, 1.359) after adjusting for all other factors.

**Table 1:** Multiple logistic regression analysis using all studies in the UK and Australia of country, year, area, age and type of study on the prevalence of wheeze

**Variable**	**All studies Odds Ratio (95% CI)(+p-value)**	**ISAAC studies only Odds Ratio (95% CI)(+p-value)**	**Non-ISAAC studies only Odds Ratio (95% CI)(+p-value)**	**ISAAC question only Odds Ratio (95% CI)(+p-value)**
Country: UK (reference) vs Australia	1.343 [1.318, 1.369] p < 0.0005	0.987 [0.958, 1.016] p = 0.373	1.470 [1.438, 1.502] p < 0.0005	0.986 [0.957, 1.017] p = 0.370
Year of survey	1.025 [1.023, 1.026] p < 0.0005	0.975 [0.971, 0.979] p < 0.0005	1.026 [1.024, 1.028] p < 0.0005	0.977 [0.973, 0.981] p < 0.0005
Age of children surveyed	0.993 [0.989, 0.997] p < 0.0005	1.007 [0.998, 1.016] p = 0.124	0.989 [0.985, 0.993] p < 0.0005	1.006 [0.995, 1.017] p = 0.260
Parental- (reference) vs Self-response	1.290 [1.246, 1.335] p < 0.0005	§ -	1.242 [1.197, 1.287] p < 0.0005	1.739 [1.624, 1.862] p < 0.0005
ISAAC study: No (reference) vs Yes	1.638 [1.598, 1.678] p < 0.0005	- - -	- - -	- - -

§OR not given as age of child (7–8 & 13–14 yrs) in ISAAC is parent-report & self-report respectively

When analysis was restricted to ISAAC studies there was a statistically significant decrease in the prevalence per survey year (2.5% per year; 95% CI 2.1%, 2.9%), after allowing for age of child and country (neither of which were significant).

If the analysis is restricted to non-ISAAC studies, even if the study had used the ISAAC question, then there was a statistically significant increase in prevalence of wheezing in Australia compared with the UK [OR = 1.470; 95% CI 1.438, 1.502], a significant increase in the prevalence per survey year (2.6% per year; 95% CI 2.4%, 2.8%), a significant decrease in the prevalence per age of child (1.1% per year; 95% CI 0.7%, 1.5%), and a significant increase in wheezing if the questionnaire was self-report compared with parental-report [OR = 1.242; 95% CI 1.197, 1.287]. This shows that the results of ISAAC studies are very different from non-ISAAC studies.

The majority of studies carried out since ISAAC phase I, have used the ISAAC question on wheezing, even if the study was not a phase I or phase III ISAAC study. So the analysis was then restricted only to studies which had used the ISAAC question on wheezing. The results showed that there was no significant difference in the prevalence of wheezing between UK and Australia and age of child was also not significant, after controlling for year of survey and parental- or self-report questions. However there has been a statistically significant decrease in the prevalence per survey year (2.3% per year; 95% CI 1.9%, 2.7%) and a significant increase in reported wheezing if the response was self- compared with parental-report (OR = 1.739; 95% CI 1.624, 1.862).

As the rate of increase in the trends for wheezing prevalence over time looked different in UK and Australia (Figure 1), an extra interaction terms was fitted in the multiple logistic regression model using all studies (Table 2). The country by year interaction effect shows that Australia has a significantly lower rate of increase in wheezing (6.2% per calendar year; 95% CI 5.7%, 6.7%) compared with the UK, after allowing for all other factors.

**Table 2:** Multiple logistic regression analysis using all studies in UK and Australia, as for Table 1, with interaction term (country × year)

**Variable**	**Odds Ratio (95% CI)**	**p-value**
Country [UK (reference) versus Australia]	1.406 [1.379, 1.433]	p < 0.0005
Year of survey	1.032 [1.030, 1.034]	p < 0.0005
Age of children surveyed	0.993 [0.989, 0.997]	p = 0.016
Parental-response (reference) vs Self-complete	1.239 [1.197, 1.283]	p < 0.0005
ISAAC study [No (reference) versus Yes]	1.649 [1.609, 1.690]	p < 0.0005
Country by Year interaction	0.938 [0.933, 0.943]	p < 0.0005

## Discussion

In this review we have reported the prevalence of current wheezing in children, published in all epidemiological studies comprising more than 1000 persons and meeting certain other quality criteria (see the Methods), over a 16-year-period, between 1990 and 2005, and further investigated the differences in reported symptoms of wheeze between ISAAC and non-ISAAC studies, in the UK and Australia.

Overall, the highest prevalence rates of current wheezing were reported in the UK, Australia and New Zealand, and the lowest prevalence was found in Albania, China, Ethiopia, Indonesia and Turkey, which gives an indication of the difference between developed and developing countries. The pattern in Africa and Asia also supports this. However, this is not supported in America, where Chile, Costa Rica and Peru had equally high wheezing prevalence as the US and Canada. Chile and Costa Rica are relatively developed countries, that may have similar characteristics in relation to development of wheezing in childhood as fully developed countries such as the US and Canada. However, this apparent inconsistency (in relation to Peru) requires further research.

Within the UK there was slightly higher prevalence of wheezing in adolescents in Scotland compared with England but there were no other substantial geographic variations, suggesting no major impact of climate, diet or outdoor environment [42]. Also, prevalence of wheezing was lower in children born outside the UK but currently residing in the UK, suggesting a role of the environment in infancy and possibly heritable genetic factors [42,43]. However, although genetic factors are important risk factors for individuals with symptoms of asthma, migrant studies indicate that they are unlikely to be responsible for the large variations in asthma symptoms that exist between populations, and cannot be responsible for the increasing prevalence of asthma within populations [44]. Environmental factors are likely to be more important and offer the greatest opportunities for prevention.

The cross-sectional ISAAC phase I study, carried out in 1994–1995, was a major achievement, and repeated in 2002–2003, the phase III study [15,16]. However, the selected ISAAC centres were most commonly an urban area (a city) and therefore may not be representative of the country. This is illustrated, for example, in Brazil where in the ISAAC phase I centres, which were all major cities, the prevalence of current wheezing in 6–7 year olds was higher than in non-ISAAC centres, which were iron-mining cities and mountainous regions (23% vs 14% respectively). The ISAAC studies also have the disadvantage of reporting wheezing symptoms only amongst two age groups (6–7 yr and 13–14 yr), and at only 2 time points (phase I and III), whereas this review shows the results of all studies of all age groups.

This review has shown that differing rates of asthma symptoms are observed in developed and developing countries. The validity of the question on wheezing in the questionnaire is likely to have varied across cultures as some languages do not have an equivalent word for "wheezing" as understood by English speakers. However, large variations in the prevalence of wheezing across the countries and over time, found in these studies are unlikely to be explained by methodological factors alone. When making comparisons of the prevalence of wheeze or asthma between different studies, it is necessary to critically assess the content of the question. There is, as yet, no accepted definition of asthma and identification of asthma by questionnaire remains a contentious issue [45]. One question is whether the everyday meaning of the word wheezing has changed over time. Do better educated parents use this word more freely for symptoms in their children? The threshold of observing mild respiratory symptoms could be lower now than previously and health campaigns may have increased parental awareness of symptoms in their children. Another interesting hypothesis is the loss of protective effect of respiratory infection in early childhood, the "hygiene hypothesis" [46]. This confirms the importance of the ISAAC phase II data collection, which was completed in 2003, and in which objective measures of pulmonary function and bronchial responsiveness are recorded in conjunction with other factors, so that further study of possible aetiological factors common to different countries can be investigated.

Australia and the UK had the most published studies on wheezing prevalence in children and were investigated in much more detail (Tables 1, 2). In addition, in these countries the ISAAC studies reported significantly higher wheezing prevalence than non-ISAAC studies and this was not due to an increase over time, nor age of child, both of which (and various other variables) were adjusted for in the analyses. It is possible that this is due to the selection of the ISAAC centres, which were all major cities. There are various possible explanations for the differences in current prevalence of wheezing including susceptibility to environmental stimuli and changes in exposure to environmental factors, genetic susceptibility, diet, low birth weight and young maternal age [47-52].

Using all the studies in UK and Australia, we find that there was a significantly higher odds of wheezing in Australia than the UK, but the rate of increase in Australia is significantly lower than the UK, as shown by the highly significant interaction between country and year. The multiple logistic regression analysis we performed for the prevalence of wheezing adjusted for age, time period, type of study (ISAAC vs non-ISAAC) and type of response (parental or self report) and does not appear therefore to result from methodological bias (e.g., confounding by ISAAC status). These differences indicate some significant discrepancy in early life environment between the two countries over the last 20 or so years.

If only ISAAC studies are investigated then there was no difference in prevalence between the two countries, after adjusting for time and age of the child, whereas non-ISAAC studies show significantly higher odds of wheezing in Australia. This is very likely because the ISAAC studies in these countries were carried out at two similar time points and for two age groups. The non-ISAAC studies span a much larger time period, use a wider range of ages and include many more study groups, and in particular are not restricted to large conurbations.

If time trends are explored in all studies in the UK and Australia, then overall there is a significant increase in the odds of wheezing over time, but only ISAAC studies show a significant decrease, which was also reported in the recent results of the phase I in 1995 and phase III in 2002, ISAAC study comparisons [16]. This is almost certainly a result of the different time periods covered by these two sorts of survey. The ISAAC studies cover the period from the early 1990s onwards, whereas other studies tend to cover earlier years, some as early as 1975. This is consistent with the trend of wheezing prevalence increasing since the 1970's but levelling out in the most recent 10–15 years, as shown in Figure 1. This is also confirmed in other reports; evidence from many repeat surveys shows that the prevalence has increased over the past 3 decades [53], but in studies between 1991 to 1998 the increase was confined to minor symptoms of asthma [54].

A decrease in reported symptoms of wheezing, as the child gets older, was observed in the UK and Australia, which confirms previous reports [55]. In ISAAC studies the age effect is not significant, perhaps because only two age groups were studied. Self-report of wheezing was significantly higher than parental-report, in the UK and Australia, again confirming previous work [56].

The ISAAC questionnaire has become almost ubiquitous since the early 1990's and we have shown that using an ISAAC question or the results of the ISAAC studies give similar results within Australia and the UK.

Wheeze may indicate undiagnosed asthma in some patients. Some studies have shown that self-reported wheeze has reasonably good specificity and sensitivity for bronchial hyper-responsiveness both in children and adults [3-5]. Wheeze is rarely a symptom of emphysema or chronic bronchitis in children, but it is very often indicative of acute viral infection, which is common in this age group [6]. Doctor-diagnosed asthma tends to be reported in only a small proportion (about 40%) of persons reporting wheeze [57], so that the possibility of selection or information bias in studies of asthma or wheeze cannot be discounted in general.

## Conclusion

In summary, the strength of this review is the reporting of the prevalence of all studies of more than 1000 persons, providing a full description of the scale and distribution of asthma symptoms (wheeze in the past year), worldwide and over time within each country. Among the countries surveyed, the UK has the highest recorded prevalence of wheezing and Ethiopia the lowest. We have documented a clear increase in the prevalence over time within Australia and the UK, with a levelling off or even decline in prevalence in more recent years. ISAAC studies show higher prevalence rates of wheezing than non-ISAAC studies.

## Competing interests

The authors declare that they have no competing interests.

## Authors' contributions

SP performed database searches, MPL determined and oversaw the statistical analyses, and all three authors contributed substantially to the conception, design and writing of the paper. All authors have read and approved the final manuscript.

## Supplementary Material

Additional file 1 **Studies of wheeze prevalence in North and South America.** The table provides the prevalence of current wheezing (in the past year), with confidence intervals, age of the children/adolescents in the study, whether the response was from the parent or the child, sample size with response rates, the year in which the study was conducted and the geographical region for each country, for all studies published between 1990 and 2005. Click here for file

Additional file 2 **Studies of wheeze prevalence in Europe.** As in Additional file 1. Click here for file

Additional file 3 **Studies of wheeze prevalence in the Eastern Mediterranean and Africa.** As in Additional file 1. Click here for file

Additional file 4 **Studies of wheeze prevalence in Asia.** As in Additional file 1. Click here for file

Additional file 5 **Studies of wheeze prevalence in Australasia.** As in Additional file 1. Click here for file
