# Supplementary material for: Systematic review of worldwide variations of the prevalence of wheezing symptoms in children
Source: Environ Health. 2008 Nov 10;7:57. doi: 10.1186/1476-069X-7-57 (PMC2614981; doi:10.1186/1476-069X-7-57)
Supplement: Additional file 1 — Studies of wheeze prevalence in North and South America. The table provides the prevalence of current wheezing (in the past year), with confidence intervals, age of the children/adolescents in the study, whether the response was from the parent or the child, sample size with response rates, the year in which the study was conducted and the geographical region for each country, for all studies published between 1990 and 2005. [file 1476-069X-7-57-S1.doc]

**Additional File 1. Studies of wheeze prevalence in North and South America**

| **Country** | **Reference** | **Survey Year** | **Area** | **N (Response rate)** | **Age (years) / ascertainment**  (P=Parental-report  S=Self-report) | **Prevalence**  **%** | **95% CI** |
| --- | --- | --- | --- | --- | --- | --- | --- |
| **Argentina** | [1] | ’94-‘95 | Buenos Aires, Rosario | 6,012^†^ (>80%):  5,374 (89.5%): | 6-7: P  13-14: S | 16.4 ^IS^  10.9 ^IS^ | 15.5, 17.3^*^  10.1, 11.7^*^ |
| **Brazil** | [2] | ‘94 | Santa Maria & Itabira | 2,714 (77%):  2,468 (77%): | 7-8: P  13-14: P | 14.3 ^IQ^  9.3 ^IQ^ | 13.0, 15.6  8.2, 10.4 |
|  | [1] | ’94-‘95:  ’95-‘96: | Porto Alegre, Recife, Sao Paulo:  Curitiba, Porto Alegre, Recife, Salvador, Sao Paulo: | 7,261^†^ (>80%):  14,697 (95.1%): | 6-7: P  13-14: S | 23.3 ^IS^  22.7 ^IS^ | 22.3, 24.3^*^  22.0, 23.4^*^ |
|  | [3] | ’95-‘96 | Sao Paulo | 1,132 (96%) | 0.5-5 P | 12.5 ^IQ^ | 10.6, 14.4^*^ |
|  | [4] | ‘99 | Sao Paulo | 3,003:  3,487: | 6-7: P  13-14: S | 24.3 ^IS^  22.1 ^IS^ | 22.8, 25.8^*^  20.7, 23.5^*^ |
| **Canada** | [1] | ’94:  ’93-‘94: | Hamilton:  Saskatoon: | 5,755 (75.1%):  4,952 (68.6%): | 6-7: P  13-14: S  6-7: P  13-14: S | 20.1 ^IS^  30.6 ^IS^  14.1 ^IS^  24.0 ^IS^ | 18.8, 21.5  29.0, 32.2  12.7, 15.4  22.1, 26.0 |
|  | [5] | Published ’95 | Montreal | 1,111 (83.9%) | 6-12 P | 5.1 ^IQ^ | 3.8, 6.4^*^ |
| **Caribbean** | [6] | ‘02 | Trinidad & Tobago | 4,988 (87.8%) | 11-19 S | 13.2 ^IQ^ | 12.3, 14.1^*^ |
| **Chile** | [7] | ’90 | La Serena | 1,730 (71%):  1,820 (71%):  1,851 (71%): | 7: P  12: P  15: P | 26.2 ^A^  20.6 ^A^  17.2 ^A^ | 25.0, 27.5  18.5, 22.7  15.7, 18.8 |
|  | [1] | ’94-‘95 | Central Santiago, Punta Arenas, South Santiago, Valdivia | 10,838^†^(>80%):  11,780(92.7%): | 6-7: P  13-14: S | 17.9 ^IS^  10.2 ^IS^ | 17.2, 18.6^*^  9.7, 10.7^*^ |
| **Costa Rica** | [1] | ’94-‘95 | Nationwide | 2,942^†^ (>80%):  2,925 (>80%): | 6-7: P  13-14: S | 32.1 ^IS^  23.7 ^IS^ | 30.4, 33.8^*^  22.2, 25.2^*^ |
|  | [8] | ‘98 | Nationwide | 1,105 (74%) | 10 P | 27.1 ^IQ^ | 24.5, 29.7^*^ |
| **Ecuador** | [9] | Published ‘03 | Pichincha & Esmeraldas | 4,433 (96.3%) | 5-18 P | 10.3 ^IQ^ | 8.8, 11.9 |
| **Mexico** | [1] | ’94-‘95 | Cuernavaca | 3,097^†^ (>80%):  2,863 (92.3%): | 6-7: P  13-14: S | 8.6 ^IS^  6.6 ^IS^ | 7.6, 9.6^*^  5.7, 7.5^*^ |
|  | [10] | Published ‘03 | Ciudad Juarez, Chihuahua | 3,390 (92%):  2,784 (92%): | 6-8: P  11-14: P | 9.7 ^IQ^  5.8 ^IQ^ | 8.7, 11.0  5.0, 6.8 |
| **Panama** | [11] | ’94-‘95 | David-Panama | 3,043^†^ (>80%):  2,775 (96.2): | 6-7: P  13-14: S | 23.5 ^IS^  17.6 ^IS^ | 22.0, 25.0^*^  16.2, 19.0^*^ |
| **Paraguay** | [1] | ’94-‘95 | Asuncion | 2,764 (93.2%) | 13-14 P | 19.4 ^IS^ | 18.0, 20.8^*^ |
| **Peru** | [1] | ’94-‘95 | Lima | 3,051 (96.6%) | 13-14 S | 26.0 ^IS^ | 24.5, 27.5^*^ |
| **Uruguay** | [1] | 94-‘95 | Montevideo | 3,071^†^ (>80%)  2,860 (93.1%) | 6-7: P  13-14: S | 18.0 ^IS^  19.0 ^IS^ | 16.6, 19.4^*^  17.6, 20.4^*^ |
| **USA** | [11] | ’88-‘94 | Nationwide | 2,318:  2,070:  1,545:  1,605: | 2-3: P  4-5: P  6-8: P  9-11: P | 26.4 ^IQ^  19.4 ^IQ^  19.2 ^IQ^  13.4 ^IQ^ | 24.6, 28.2^*^  17.7, 21.1^*^  17.2, 21.1^*^  11.7, 15.0^*^ |
|  | [12] | ’94 | Illinois | 2,693 (90%) | 13-18 S | 25.1 ^IQ^ | 23.5, 26.7^*^ |
|  | [13] | ’94-‘95 | Seattle | 1,865 (37%) | 5-9 P | 18.8 ^B^ | 17.0, 20.6^*^ |
|  | [1] | ’94-‘95 | Chicago, Seattle | 6,660 (88.7%) | 13-14 S | 21.7 ^IS^ | 20.8, 22.6^*^ |
|  | [14] | ’97 | New York City – East Harlem | 1,139 (82%) | 5-12 P | 24.5 ^IQ^ | 22.0, 27.0^*^ |
|  | [15] | Published ’00 | North Carolina | 1,596 (92%) | 13-14 S | 21.0 ^IQ^ | 19.0, 23.0^*^ |
|  | [16] | ’99-‘00 | North Carolina | 128,568 (88%) | 13-14 S | 26.1 ^IQ^ | 25.9, 26.3^*^ |
|  | [17] | ‘00 | New Orleans | 1,535 | 5-18 P/S | 25.7 ^IQ^ | 23.5, 27.9^*^ |
|  | [18] | ‘01 | South Plains & Panhandle, West Texas | 1,500 (64%) | <16 P | 17.5 ^IQ^ | 15.6, 19.4^*^ |
|  | [19] | ’00-‘02 | Iowa | 3,090 (86.6%) | 6-14 P | 19.1 ^IQ^ | 17.7, 20.5^*^ |

Key:

‘Prevalence’

IS: ISAAC study, with question “Have you had wheezing and whistling in the chest in the last 12 months?” (Yes/No)

IQ: ISAAC question, but not an ISAAC study

A: In the past 12 months has your child had a wheezing or asthma attack? (Yes/No)

B: Current wheezing without a diagnosis of asthma & Physician diagnosed asthma

C: In the last 12 months, has a wheeze (that is, a whistling noise, high or low pitched) ever been heard from your child’s chest?

D: Has your child (ever) wheezed in the past 12 months?

E: Wheeze in the previous year (interview questionnaire)

F: Have you had wheezing attacks in the past year?

G: Has your child had wheezing in the chest (but not from the throat or nose)

H: Wheezy or whistling sound in the chest when having a cold or occasionally apart from colds or for most days or nights, in the past 12 months

^*^ CI not given in the publication and calculated by author

† N is the number of questionnaires given out & response rate obtained from ISAAC study [1,20]

**References**

1. ISAAC Steering Committee. Worldwide variations in the prevalence of asthma symptoms: the International Study of Asthma and Allergies in Childhood (ISAAC). *European Respiratory Journal* 1998;12:315-35.

2. Werneck G, Ruiz S, Hart R, White M, Romieu I. Prevalence of asthma and other childhood allergies in Brazilian schoolchildren. *Journal of Asthma* 1999;36:677-90.

3. Benicio MHD, Ferreira MU, Cardoso MRA, Konno SC, Monteiro CA. Wheezing conditions in early childhood: prevalence and risk factors in the city of Sao Paulo, Brazil. *Bulletin of The World Health Organization* 2004;82(7):516-22.

4. Sole D, Camelo-Nunes IC, Wandalsen GF, Melo KC, Naspitz CK. Is rhinitis alone or associated with atopic eczema a risk factor for severe asthma in children? *Pediatric Allergy and Immunology* 2005;16(2):121-125.

5. Ernst P, Demissie K, Joseph L, Locher U, Becklake MR. Socioeconomic status and indicators of asthma in children. *American Journal of Respiratory and Critical Care Medicine* 1995;152:570-5.

6. Monteil MA, Joseph G, Changkit C, Wheeler G, Antoine RM. Comparison of prevalence and severity of asthma among adolescents in the Caribbean islands of Trinidad and Tobago: results of a nationwide cross-sectional survey. *BMC Public Health* 2005;5:96.

7. Robertson CF, Bishop J, Sennhauser FH, Mallol J. International comparison of asthma prevalence in children: Australia, Switzerland, Chile. *Pediatric Pulmonology* 1993;16:219-26.

8. Soto-Quiros ME, Soto-Martinez M, Hanson LA. Epidemiological studies of the very high prevalence of asthma and related symptoms among school children in Costa Rica from 1989 to 1998. *Pediatric Allergy and Immunology* 2002;13:342-9.

9. Cooper PJ, Chico ME, Bland M, Griffin GE, Nutman TB. Allergic symptoms, atopy and Geohelminth infections in a rural area of equador. *American Journal of Respiratory and Critical Care Medicine* 2003;168:313-7.

10. Villarreal AB, Aguirre LHS, Rojo MMT, Navarro ML, Romieu I. Risk factors for asthma in school children from Ciudad Juarez, Chihauhau. *Journal of Asthma* 2003;40(4):413-23.

11. Eldeirawi K, Persky VW. History of ear infections and prevalence of asthma in a national sample of children aged 2 to 11 years: the Third National Health and Nutrition Examination Survey, 1988 to 1994. *Chest* 2004;125(5):1685-92.

12. Fagan JK, Scheff PA, Hryhorczuk D, Ramakrishnan V, Ross M, Persky V. Prevalence of asthma and other allergic diseases in an adolescent population: association with gender and race. *Annals of Allergy, Asthma and Immunology* 2001;86:177-84.

13. Maier WC, Arrighi HM, Morray B, Llewllyn C, Redding GJ. The impact of asthma and asthma-like illness in Seattle school children. *Journal of Clinical Epidemiology* 1998;51:557-68.

14. Findley S, Lawler K, Bindra M, Maggio L, Penachio MM, Maylahn C. Elevated asthma and indoor environmental exposures among Puerto Rican children of East Harlem. *Journal of Asthma* 2003;40(5):557-69.

15. Yeatts K, Shy C, Wiley J, Music S. Statewide adolescent asthma surveillance. *Journal of Asthma* 2000;37:425-34.

16. Sotir M, Yeatts K, Shy C. Presence of asthma risk factors and environmental exposures related to upper respiratory infection-triggered wheezing in middle school-age children. *Environmental Health Perspectives* 2003;111:657-62.

17. Mvula M, Larzelere M, Kraus M, Moisiewicz K, Morgan C, Pierce S, Post R, Nash T, Moore C. Prevalence of asthma and asthma-like symptoms in inner-city schoolchildren. *Journal of Asthma* 2005;42(1):9-16.

18. Arif AA, Borders TF, Patterson PJ, Rohrer JE, Xu KT. Prevalence and correlates of paediatric asthma and wheezing in a largely rural USA population. *Journal of Paediatrics and Child Health* 2004;40(4):189-94.

19. Chrischilles E, Ahrens R, Kuehl A, Kelly K, Thorne P, Burmeister L, Merchant J. Asthma prevalence and morbidity among rural Iowa schoolchildren. *Journal of Allergy and Clinical Immunology* 2004;113(1):66-71.

20. ISAAC Steering Committee. Worldwide variation in prevalence symptoms of asthma, allergic rhinoconjunctivitis and atopic eczema: ISAAC. *Lancet* 1998;351:1225-32.
