# Supplementary material for: Systematic review of worldwide variations of the prevalence of wheezing symptoms in children
Source: Environ Health. 2008 Nov 10;7:57. doi: 10.1186/1476-069X-7-57 (PMC2614981; doi:10.1186/1476-069X-7-57)
Supplement: Additional file 2 — Studies of wheeze prevalence in Europe. As in Additional file 1. [file 1476-069X-7-57-S2.doc]

**Additional File 2. Studies of wheeze prevalence in Europe**

| **Country** | **Reference** | **Survey Year** | **Area** | **N (Response rate)** | **Age (years) / ascertainment**  (P=Parental-report  S=Self-report) | **Prevalence**  **%** | **95% CI** |
| --- | --- | --- | --- | --- | --- | --- | --- |
| **Albania** | [1] | ’94-‘95 | Tirane | 2,981^†^ (>80%):  2,862 (96.8%): | 6-7: P  13-14: S | 7.6 ^IS^  2.6 ^IS^ | 6.6, 8.6^*^  2.0, 3.2^*^ |
| **Austria** | [1] | ’94-‘95 | Salzburg, Urfahr-Umgebung | 5,787^†^ (>80%)  4,270 (87.40%) | 6-7: P  13-14: S | 8.9 ^IS^  11.6 ^IS^ | 8.2, 9.6^*^  10.7, 12.5^*^ |
|  | [2] | ’97 | Salzburg | 2,283 (95.4%) | 8-10 P | 7.1 ^IQ^ | 6.0, 8.2^*^ |
| **Belgium** | [1] | ’94-‘95:  ‘95-‘96: | Antwerp | 5,782 (88.5%):  1,477 (97.5%): | 6-7: P  13-14: S | 7.3 ^IS^  12.0 ^IS^ | 6.6, 8.0^*^  10.3, 13.7^*^ |
| **Bulgaria** | [3] | ‘96 | Four urban areas | 3,631 (92%) | 7-11 P | 14.5 ^IQ^ | 13.4, 15.6^*^ |
| **Czech Republic** | [3] | ‘96 | Four urban areas | 3,479 (55%) | 7-11 P | 14.7 ^IQ^ | 13.5, 15.9^*^ |
| **Cyprus** | [4] | ’97 | Northern Cyprus | 2,529 (89.6%) | 6-14 P | 4.8 ^IQ^ | 4.0, 5.6 |
| **Estonia** | [5] | ’92-3 | Tallin & Tartu | 1,519 (96.1%) | 10-12 P | 7.0 ^C^ | 5.7, 8.3^*^ |
|  | [6] | ’93-’94:  ’01-’02: | Tallinn | 3,070 (90.8%):  3,506 (88.7%):  2,388 (85.7%):  3,605 (93.4%): | 6-7: P  13-14: S  6-7: P  13-14: S | 9.3 ^IS^  8.5  ^IS^  9.7 ^IS^  9.2 ^IS^ | 8.3, 10.3^*^  7.6, 9.4^*^  8.5, 10.9^*^  8.3, 10.1^*^ |
| **Finland** | [1] | ’93 | Kuopio | 2,564 (86%) | 7-12 P | 5.4 ^D^ | 4.5, 6.3^*^ |
|  | [1] | ’94-‘95 | Lapland, Kuopio, Turku & Pori:  Helsinki: | 8,836:  2,771: | 13-14 S | 14.7 ^IS^  19.8 ^IS^ | 14.0, 15.4^*^  18.3, 21.3^*^ |
| **France** | [1] | ’94-‘95 | Pessac:  Marseilles, Montpellier, Pessac, Strasbourg, West Marne: | 3,202^†^ (>80%):  15,410 (83.1%): | 6-7: P    13-14: S | 8.1 ^IS^  13.5 ^IS^ | 7.2, 9.0^*^  13.0, 14.0^*^ |
|  | [7] | ’99-‘00 | Bordeaux, Clermont-Ferrand, Creteil, Marseille, Strasbourg, Reims | 4,901 (80.9%) | 9-11 P | 8.0 ^IQ^ | 7.2, 8.8^*^ |
| **Georgia** | [1] | ’94-‘95 | Kutaisi, Tbilisi | 6,770^†^ (>80%):  6,098 (90.4%): | 6-7: P  13-14: S | 7.6 ^IS^  3.6 ^IS^ | 7.0, 8.2^*^  3.2, 4.0^*^ |
| **Germany** | [8] | ’91 | Bochum | 1,928 (93%) | 12-15 S | 20.0 ^IQ^ | 18.2, 21.8^*^ |
|  | [1] | ’94-‘95 | Munster (W Germany)  Greifswald (E Germany) | 3,036 (81.2%):  3,763 (94.0%):  2,451 (85.9%):  2,776 (87.6%): | 6-7: P  13-14: S  6-7: P  13-14: S | 9.6 ^IS^  14.1 ^IS^  7.2 ^IS^  13.3 ^IS^ | 8.6, 10.6^*^  13.6, 15.8^*^  6.2, 8.2^*^  12.0, 14.6^*^ |
|  | [9] | ’95-‘96 | E Germany (Leipzig, Dresden) :  W Germany (Munich): | 9,524 (84.3%):  4,777: | 5-11: P  5-11: P | 7.7 ^IQ^  8.8 ^IQ^ | 7.2, 8.2^*^  8.0, 9.6^*^ |
|  | [10] | ’95-‘96 | Zerbst, Bitterfeld, Hettstedt | 2,814 (74.7%) | 5-14 P | 24.8 ^IQ^ | 23.1, 26.5^*^ |
|  | [11] | ’99-‘00 | Munster (W Germany) | 3,529 (82%):  3,816 (94%): | 6-7: P  13-14: S | 13.2 ^IS^  17.5 ^IS^ | 2.1, 14.3^*^  16.3, 18.7^*^ |
| **Greece** | [1] | ’94-‘95 | Athens | 1,654^†^ (>80%)  2,228 (87.0%) | 6-7: P  13-14: S | 7.6 ^IS^  3.7 ^IS^ | 6.3, 8.9^*^  3.0, 4.4^*^ |
| **Hungary** | [3] | ‘96 | Five urban areas | 3,721 (66%) | 7-11 P | 6.5 ^IQ^ | 5.7, 7.3^*^ |
| **Ireland** | [12] | ’92-‘93 | Dublin, Wicklow, Kildare | 1,416:  1,317: | 4-10: P  11-19: P | 16.5 ^C^  14.9 ^C^ | 14.6, 18.4^*^  13.0, 16.8^*^ |
|  | [1] | ’95 | Nationwide | 2,898 (92.1%) | 13-14 S | 29.1 ^IS^ | 27.4, 30.8 |
|  | [13] | ’00 | Counties Louth & Mead | 1,899 (61.4%) | 6-7 P | 17.4 ^IQ^ | 15.7, 19.1^*^ |
| **Italy** | [1] | ’93-‘95 | Northern & Central Italy | 19,982 (96%):  25,497 (96.3%): | 6-7: P  13-14: S | 7.3 ^IS^  8.9 ^IS^ | 6.9, 7.7^*^  8.6, 9.2^*^ |
|  | [14] | ‘02 | North, Central & South Italy | 20,016 (89%):  13,266 (81%): | 6-8: P  13-14: P+S | 7.9 ^IQ^  8.3 ^IQ^ | 7.5, 8.3^*^  7.8, 8.8^*^ |
| **Latvia** | [1] | ’94-‘95 | Riga:  Riga, Rural Latvia: | 3,003^†^ (>80%):  5,755 (93.6%): | 6-7: P  13-14: S | 7.3 ^IS^  8.4 ^IS^ | 6.4, 8.2^*^  7.7, 9.1^*^ |
| **Malta** | [15] | ’95 | Nationwide | 4,184 (88.7%) | 13-15 S | 16.0 ^IS^ | 14.9, 17.1^*^ |
|  | [16] | Published ‘02 | Malta and Gozo | 3,506 (78.5%) | 5-8 P | 8.8 ^IS^ | 7.9, 9.7^*^ |
| **Norway** | [17] | Published ‘97 | Oslo, Hallingdal, Odda | 924 (88%):  1,877 (88%):  1,213 (88%): | 6-8: P  9-12: P  13-16: P | 13.6 ^IQ^  11.4 ^IQ^  12.2 ^IQ^ | 11.4, 15.8  10.0, 12.8  10.4, 14.0 |
|  | [18] | ’95-‘97 | Nord-Trondelag County | 8,571 (86%) | 13-19 S | 26.1 ^IQ^ | 25.2, 27.0^*^ |
| **Poland** | [1] | ’93-‘95 | Krakow, Poznan | 4,974^†^ (>80%):  9,282 (91.3%): | 6-7: P  13-14: S | 10.9 ^IS^  8.1 ^IS^ | 10.0, 11.8^*^  7.6, 8.6^*^ |
|  | [3] | ‘96 | Four urban areas | 2,932 (66%) | 7-11 P | 9.6. ^IQ^ | 8.5, 10.7^*^ |
|  | [19] | ’96-‘98 | Swietochlowice, Kedzierzyn-Kozle, Pszczyna & Kielce | 1,561 (80%) | 9-11 P | 8.6 ^D^ | 7.2, 10.0^*^ |
| **Portugal** | [1] | ‘96 | Funchal, Lisbon, Portimao:  Funchal, Lisbon, Portimao, Porto: | 5,129^†^ (>80%):  9,977 (92.8%): | 6-7: P  13-14: S | 13.2 ^IS^  9.5 ^IS^ | 12.4, 14.2^*^  8.9, 10.1^*^ |
| **Romania** | [1] | ’94-‘95 | Cluj | 3,362 (99.0%) | 13-14 S | 3.0 ^IS^ | 2.4, 3.6^*^ |
|  | [3] | ‘96 | Four urban areas | 3,470 (66%) | 7-11 P | 6.4 ^IQ^ | 5.6, 7.2^*^ |
| **Russia** | [1] | ’94-‘95 | Moscow | 2,910 (85.3%) | 13-14 S | 4.4 ^IS^ | 3.7, 5.1^*^ |
|  | [20] | Published ‘04 | 9 cities | 5,951 (98%) | 8-12 P | 13.4 ^IQ^ | 12.5, 14.3^*^ |
| **Slovak Republic** | [3] | ‘96 | Four urban areas | 3,038 (66%) | 7-11 P | 4.5 ^IQ^ | 3.8, 5.2^*^ |
| **Spain** | [1] | ’94-‘95 | 5 cities:  8 cities: | 16,884^†^ (>80%):  23,770 (95.0%): | 6-7: P  13-14: S | 6.2 ^IS^  10.3 ^IS^ | 5.8, 6.6^*^  9.9, 10.7^*^ |
|  | [21] | ’02-‘03 | 11 cities | 18,599 (71.5%):  24,214 (87.3%): | 6-7: P  13-14: S | 9.4 ^IS^  9.2 ^IS^ | 9.0, 9.8^*^  8.8, 9.6^*^ |
|  | [22] | Published ‘05 | 3 cities | 2,720 (33.8%) | 9-12 P ^IQ^ | 13.1 ^IQ^ | 11.8, 14.4^*^ |
| **Sweden** | [23] | ‘90 | Norrkoping | 1,276 (90.4%) | 12 P | 9.2 ^D^ | 7.6, 10.8^*^ |
|  | [1] | ’94-‘95 | Stockholm, Uppsala:  Linkoping, Stockholm, Uppsala: | 3,029^†^ (>80%)  6,039 (93.6%) | 6-7: P  13-14: S | 10.4 ^IS^  12.9  ^IS^ | 9.3, 11.5^*^  12.1, 13.7^*^ |
|  | [24] | ’91: (cohort) | Goteborg, Karuna | 2,481 (87.6%): | 7-8: P | 7.6 ^E^ | 6.6, 8.6^*^ |
|  | [25] | ’96-‘98: | Karuna, Lulea, Pitea | 3,247 (92%) | 7-10: P | 10.9 ^IQ^ | 9.8, 12.0^*^ |
|  | [26] | ’97 | Linkoping, Ostersund | 2,108 (84.2%) | 10-11 P | 9.2 ^IQ^ | 8.0, 10.4^*^ |
|  | [27] | ‘00 | Varmland | 10,851 (79%) | 1-6 P | 19.3 ^IQ^ | 18.6, 20.0^*^ |
|  | [28] | ‘02 | Lulea, Pitea, Karuna | 3,345 (95.2%) | 13-14 S | 10.5 ^IQ^ | 9.5, 11.5^*^ |
| **Switzerland** | [29] | ’90 | Canton of St Galon | 5,338 (97.8%) | 7: P  12: P  15: P | 7.4 ^A^  6.0 ^A^  4.5 ^A^ | 5.9, 8.8  4.7, 7.3  3.5, 5.6 |
|  | [30] | ’92-’93:  ’95-’97:  ’99-’00: | 4 urban & 4 rural communities | 1,324 (74.9%):  1,668 (80.6%):  1,250 (73.9%): | 13-14 S | 9.7 ^IQ^  9.2 ^IQ^  10.9 ^IQ^ | 8.1, 11.3^*^  7.8, 10.6^*^  9.2, 12.6^*^ |
|  | [31] | ’92-’93:  ’98-’01: | Urban, suburban, rural and alpine areas | 4,026:  9,591: | 6-14 P | 8.2 ^IQ^  6.1 ^IQ^ | 5.6, 11.8^*^  4.2, 8.9^*^ |
| **The Netherlands** | [32] | Published ‘92 | Helmond | 3,344 (73%) | 6-12 P | 10.1 ^C^ | 9.1, 11.1^*^ |
|  | [33] | ’91-‘92 | Nationwide (Child Health Monitoring System) | 2,677 (92%):  2,509 (92%): | 4-9: P  10-15:  (P<12 / S) | 8.9 ^D^  5.5 ^D^ | 7.8, 10.0^*^  4.6, 6.4^*^ |
|  | [34] | ’93 | Melick-Herkenbosch, Leek | 1,190 (97%) | 6-12 P | 14.4 ^D^ | 12.4, 16.4^*^ |
|  | [35] | ’89:  ’93:  ’97:  ’01: | Westelijke Mijnstreek | 1,794 (>95%):  1,526 (>95%):  1,670 (>95%):  1,102 (95.5%): | 8-9 P | 13.4 ^D^  13.3 ^D^  11.9 ^D^  9.1 ^D^ | 11.8, 15.0^*^  11.6, 15.0^*^  10.3, 13.5^*^  7.4, 10.8^*^ |
| **Uzbekistan** | [1] | ’94-‘95 | Samarkand, Tashkent | 4,354 (93.4%) | 13-14 S | 9.2 ^IS^ | 8.4, 10.0^*^ |
| **UK** | [36] | ’75:  ’80:  ’86: | Nationwide | 12,402:  13,575:  9,006: | 5: P  10: P  16: S | 9.9 ^D^  7.6 ^D^  6.6 ^D^ | 9.4, 10.4^*^  7.2, 8.0^*^  6.1, 7.1^*^ |
|  | [37] | ’87:  ’95: | Southampton | 3,187 (90%):  2,289 (75.5%): | 6-8: P  14-16: P | 14.6 ^D^  18.2 ^D^ | 13.4, 15.8^*^  16.6, 19.8^*^ |
|  | [38] | ’89 | Walsall | 1,334 (81.8%) | 6-7 P | 11.1 ^IQ^ | 9.4, 12.8^*^ |
|  | [39] | ’89-‘90 | Southampton | 1,033 (76.7%) | 7-12 P | 17.2 ^D^ | 14.9, 19.5^*^ |
|  | [40] | ’90 | Leicestershire | 1,422 (86.2%) | 0-5 P | 13.0 ^F^ | 11.3, 14.8 |
|  | [41] | ’78:  ’91: | Croydon | 4,147 (87%):  3,070 (81%): | 7.5-8.5 P | 11.1 ^F^  12.9 ^F^ | 10.1, 12.1^*^  11.7, 14.1^*^ |
|  | [8] | ’91 | West Sussex | 2,097 (92%) | 12-15 S | 29.1 ^IQ^ | 27.2, 31.0^*^ |
|  | [42] | ’91:  ’99: | Sheffield | 4,580 (85.3%):  5,011 (83.2%): | 8-9: P  8-9: P | 17.0 ^IQ^  19.4 ^IQ^ | 15.9, 18.1  18.3, 20.5 |
|  | [43] | ’92 | National | 2,866:  2,606: | 5-10: P  11-17: P | 16.3 ^IQ^  13.6 ^IQ^ | 14.9, 17.7^*^  12.3, 14.9^*^ |
|  | [44] | ’93 | East Surrey | 1,936 (97%) | 13-14 S | 24.3 ^IQ^ | 22.4, 26.2^*^ |
|  | [45] | ’88:  ’95: | Nottingham | 13,579 (78%):  22,968 (83%): | 4-11 P | 12.5 ^F^  15.1 ^F^ | 11.9, 13.1^*^  14.6, 15.6 |
|  | [46] | ’92:  ’94: | Scottish Highlands | 1,825:  1,537 (85%): | 12,14: P  12,14: P | 19.2 ^C^  17.4 ^C^ | 17.4, 21.0^*^  15.5, 19.3^*^ |
|  | [1] | ’94-‘95 | Sunderland:  Nationwide: | 1,864^†^ (>80%):  35,485 (86.9%) | 6-7: P  13-14: S | 18.4 ^IS^  32.2 ^IS^ | 16.6, 20.2^*^  31.8, 32.8^*^ |
|  | [47] | ‘96 | Nottingham | 27,826 | 11-16: S | 19.0 ^F^ | 18.5, 19.5^*^ |
|  | [48] | ’90:  ’98: | Leicestershire | 1,422 (86%):  2,127 (84%): | 1-5: P  1-5: P | 12.3 ^F^  25.4 ^F^ | 10.6, 14.0^*^  23.6, 27.2^*^ |
|  | [49] | Published ‘99 | North-East England | 3,000 (80.0%) | 6-7 P | 18.0 ^IQ^ | 16.6, 19.4 |
|  | [50] | ’99 | Sheffield | 4,311 (83.2%) | 8-9 P | 20.2 ^IQ^ | 19.0, 21.4^*^ |
|  | [51] | ’99-‘00 | Isle of Wight | 1,373 (94.3%) | 10 P | 18.9 ^IQ^ | 16.8, 21.0^*^ |
|  | [52] | ’93:  ’95:  ’99:  ’01: | Manchester | 2,478:  2,228:  1,985:  1,781: | <16 P | 27 ^IQ^  24 ^IQ^  25 ^IQ^  22 ^IQ^ | 25, 29  22, 26  23, 27  20, 24 |
|  | [53] | ’00-‘01 | Aberdeen | 1,374 (71.4%) | 2 P | 15.3 ^G^ | 13.4, 17.2^*^ |
|  | [54] | Published ‘02 | Swindon, Basingstoke, Hook, Fleet | 1,732 (56.2%) | 7-9 P | 18.9 ^IQ^ | 17.1, 20.7^*^ |
|  | [55] | ‘02 | Nationwide | 15,755 | 12-14 S | 27.5 ^IQ^ | 26.8, 28.2^*^ |
|  | [56] | ‘03 | East Midlands, Eastern Region | 11,562 | 4-6 P | 14.1 ^IQ^ | 13.5, 14.7^*^ |

Key:

‘Prevalence’

IS: ISAAC study, with question “Have you had wheezing and whistling in the chest in the last 12 months?” (Yes/No)

IQ: ISAAC question, but not an ISAAC study

A: In the past 12 months has your child had a wheezing or asthma attack? (Yes/No)

B: Current wheezing without a diagnosis of asthma & Physician diagnosed asthma

C: In the last 12 months, has a wheeze (that is, a whistling noise, high or low pitched) ever been heard from your child’s chest?

D: Has your child (ever) wheezed in the past 12 months?

E: Wheeze in the previous year (interview questionnaire)

F: Have you had wheezing attacks in the past year?

G: Has your child had wheezing in the chest (but not from the throat or nose)

H: Wheezy or whistling sound in the chest when having a cold or occasionally apart from colds or for most days or nights, in the past 12 months

^*^ CI not given in the publication and calculated by author

† N is the number of questionnaires given out & response rate obtained from ISAAC study [1,57]

**References**

1. ISAAC Steering Committee. Worldwide variations in the prevalence of asthma symptoms: the International Study of Asthma and Allergies in Childhood (ISAAC). *European Respiratory Journal* 1998;12:315-35.

2. Riedler J, Eder W, Oberfeld G, Schreuer M. Austrian children living on a farm have less hay fever, asthma and allergic sensitization. *Clinical and Experimental Allergy* 2000;30:194-200.

3. Leonardi GS, Houthuijs D, B. N, J. V, Rudnai P, Zejda J, Gurzau E, Fabianova E, Fletcher T, Brunekreef B. Respiratory syptoms, bronchitis and asthma in children of Central and Eatern Europe. *European Respiratory Journal* 2002;20:890-8.

4. Kalayci O, Saraclar Y, Sekerel BE, Adalioglu G, Kuyucu S, Egor G, Bozer HK, Tuncer A. Prevalence of asthma symptoms among Turkish Cypriot schoolchildren. *Turkish Journal of Pediatrics* 1999;41:413-20.

5. Riikjarv MA, Julge K, Vasar M, Braback L, Knutsson A, Bjorksten B. The prevalence of atopic sensitization and respiratory symptoms among Estonian schoolchildren. *Clinical and Experimental Allergy* 1995;25:1198-204.

6. Annus T, Riikjarv M-A, Rahu K, Bjorksten B. Modest increase in seasonal allergic rhinitis and eczema over 8 years among Estonian schoolchildren. *Pediatric Allergy and Immunology* 2005;16(4):315-20.

7. Penard-Morand C, Charpin D, Raherison C, Kopferschmitt C, Caillaud D, Lavaud F, Annesi-Maesano I. Long-term exposure to background air pollution related to respiratory and allergic health in schoolchildren. *Clinical and Experimental Allergy* 2005;35(10):1279-87.

8. Pearce NE, Weiland S, Keil U, Langridge P, Anderson HR, Strachan D, Bauman A, Young L, Gluyas P, Ruffin D, Crane J, Beasley R. Self-reported prevalence of asthma symptoms in children in Australia, England, Germany and New Zealand: An international comparison using the ISAAC written and video questionnaires. *European Respiratory Journal* 1993;6:1455-61.

9. Weiland SK, von Mutius E, Hirsch T, Duhme H, Fritzsch C, Werner B, Husing A, Stender M, Renz H, Leupold W, Keil U. Prevalence of respiratory and atopic disorders among children in the East and West of Germany five years after unification. *European Respiratory Journal* 1999;14:862-70.

10. Wjst M, Hoelscher B, Frye C, Wichmann HE, Dold S, Heinrich J. Early antibiotic treatment and later asthma. *European Journal of Medical Research* 2001;6:263-71.

11. Maziak W, Behrens T, Brasky TM, Duhme H, Rzehak P, Weiland SK, Keil U. Are asthma and allergies in children and adolescents increasing? Results from ISAAC phase I and phase III surveys in Munster, Germany. *Allergy* 2003;58:572-9.

12. Taylor MR, Holland CV, O'Lorcain P. Asthma and wheeze in schoolchildren. *Irish Medical Journal* 1996;89(1):34-5.

13. Harty SB, Sheridan A, Howell F, Nicholson A. Wheeze, eczema and rhinitis in 6-7 year old Irish schoolchildren. *Irish Medical Journal* 2003;96:102-4.

14. Simoni M, Lombardi E, Berti G, Rusconi F, La Grutta S, Piffer S, Petronio M, Galassi G, Forastiere F, Viegi G. Mould/dampness exposure at home is associated with respiratory disorders in Italian children and adolescents: the SIDRIA-2 Study. *Occupational and Environmental Medicine* 2005;62(9):616-22.

15. Montefort S, Lenicker HM, Caruna S, Agius-Muscat H. Asthma, rhinitis and eczema in Maltese 13-15 year-old schoolchildren - prevalence, severity and associated factors [ISAAC]. *Clinical and Experimental Allergy* 1998;28:1089-99.

16. Montefort S, Muscat HA, Caruna S, Lenicker H. Allergic conditions in 5-8 year-old Maltese schoolchildren: Prevalence, severity and associated risk factors [ISAAC]. *Pediatric Allergy and Immunology* 2002;13:98-104.

17. Nystad W, Magnus P, Roksund O, Svidal B, Hetlevik O. The prevalence of respiratory symptoms and asthma among schoolchildren in three different areas of Norway. *Pediatric Allergy and Immunology* 1997;8:35-40.

18. Henriksen AH, Holmen TL, Bjermer L. Gender differences in asthma prevalence may depend on how asthma is defined. *Respiratory Medicine* 2003;97:491-7.

19. Zlotkowska R, Zejda JE. Fetal and postnatal exposure to tobacco smoke and respiratory health in children. *European Journal of Epidemiolology* 2005;20:719-27.

20. Spengler JD, Jaakkola JJK, Parise H, Katsnelson BA, Privalova LI, Kosheleva AA. Housing characteristics and children's respiratory health in the Russian Federation. *American Journal of Public Health* 2004;94(4):657-62.

21. Garcia-Marcos L, Quiros AB, Hernandez GG, Guillen-Grima F, Diaz CG, Urena IC, Pena AA, Monge RB, Suarez-Varela MM, Varela AL-S, Cabanillas PG, Garrido JB. Stabilization of asthma prevalence among adolescents and increase among schoolchildren (ISAAC phases I and III) in Spain. *Allergy* 2004;59(12):1301-7.

22. Garcia-Marcos L, Castro-Rodriguez JA, Suarez-Varela MM, Garrido JB, Hernandez GG, Gimeno AM, Gonzalez AL, Ruiz TR, Torres AM. A different pattern of risk factors for atopic and non-atopic wheezing in 9-12-year-old children. *Pediatric Allergy and Immunology* 2005;16(6):471-7.

23. Burr ML, Limb ES, Andrae S, Barry DMJ, Nagel F. Childhood asthma in four countries: a comparative survey. *International Journal of Epidemiology* 1994;23:341-7.

24. Hesselmar B, Aberg B, Eriksson B, Aberg N. Asthma in children: prevalence, treatment and sensitization. *Pediatric Allergy and Immunology* 2000;11:74-9.

25. Ronmark E, Perzanowski M, Platts-Mills T, Lundback B. Incidence rates and risk factors for asthma among school children: A 2-year follow-up report from the Obsructive Lung Disease in Northern Sweden (OLIN) studies. *Respiratory Medicine* 2002;96:1006-13.

26. Braback L, Kjellman NI, Sandin A, Bjorksten B. Atopy among schoolchildren in northern and southern Sweden in relation to pet ownership and early life events. *Pediatric Allergy and Immunology* 2001;12:4-10.

27. Bornehag CG, Sundell J, Hagerhed L, Janson S. Pet-keeping in early childhood and airway, nose and skin symptoms later in life. *Allergy* 2003;58:939-44.

28. Hedman L, Lindgren B, Perzanowski M, Ronmark E. Agreement between parental and self-completed questionnaires about asthma in teenagers. *Pediatric Allergy and Immunology* 2005;16(2):176-81.

29. Sennhauser FH, Kuhni CE. Prevalence of respiratory symptoms in Swiss children: is bronchial asthma really more prevalent in boys? *Pediatric Pulmonology* 1995;19:161-6.

30. Braun-Fahrlander C, Gassner M, Grize L, Takken-Sahli K, Neu U, Stricker T, Varonier HS, Wuthrich B, Sennhauser FH. No further increase in asthma, hay fever and atopic sensitisation in adolescents living in Switzerland. *European Respiratory Journal* 2004;23(3):407-13.

31. Bayer-Oglesby L, Grize L, Gassner M, Takken-Sahli K, Sennhauser FH, Neu U, Schindler C, Braun-Fahrlander C. Decline of ambient air pollution levels and improved respiratory health in Swiss children. *Environmental Health Perspectives* 2005;113(11):1632-7.

32. Brunekreef B, Groot B, Hoek G. Pets, allergy and respiratory symptoms in children. *International Journal of Epidemiology* 1992;21:338-42.

33. Spee-van-der-Wekke J, Meulmeester JF, Radder JJ, Verloove-Vanhorick SP. School absence and treatment in schoolchildren with respiratory symptoms in The Netherlands: data from the Child Health Monitoring System. *Journal of Epidemiology and Community Health* 1998;52:359-63.

34. de-Kok ME, Mertens PL, Cuijpers CE, Swaen GM, Wesseling GJ, Broer J, Sturmans F, Wouters EFM. The rate of respiratory symptoms among primary schoolchildren in two Dutch regions. *European Journal of Pediatrics* 1996;155(6):506-11.

35. Mommers M, Gielkens-Sijstermans C, Swaen GMH, van Schayck CP. Trends in the prevalence of respiratory symptoms and treatment in Dutch children over a 12 year period: results of the fourth consecutive survey. *Thorax* 2005;60(2):97-9.

36. Lewis S, Richards D, Bynner J, Butler N, Britton J. Prospective study of risk factors for early and persistent wheezing in childhood. *European Respiratory Journal* 1995;8:349-56.

37. Withers NJ, Low L, Holgate ST, Clough JB. The natural history of respiratory symptoms in a cohort of adolescents. *American Journal of Respiratory and Critical Care Medicine* 1998;158:352-7.

38. Symington P, Coggon D, Holgate S. Respiratory symptoms in children at schools near a foundry. *British Journal of Industrial Medicine* 1991;48:588-91.

39. Pararajasingam CD, Sittampalam L, Damani P, Pattemore PK, Holgate ST. Comparison of the prevalence of asthma among Asian and European children in Southampton. *Thorax* 1992;47:529-32.

40. Luyt DK, Burton PR, Simpson H. Epidemiological study of wheeze, doctor diagnosed asthma and cough in preschool children in Leicestershire. *British Medical Journal* 1993;306:1386-90.

41. Anderson HR, Butland BK, Strachan DP. Trends in prevalence and severity of childhood asthma. *British Medical Journal* 1994;308:1600-4.

42. Ng-Man-Kwong G, Proctor A, Billings C, Duggan R, Das C, Whyte MK, Powell CVE, Primhak R. Increasing prevalence of asthma diagnosis and symptoms in children is confined to mild symptoms. *Thorax* 2001;56:312-4.

43. Strachan DP, Anderson HR, Limb ES, O'Neill A, Wells N. A national survey of asthma prevalence, severity and treatment in Great Britain. *Archives of Disease in Childhood* 1994;70:174-8.

44. Waldron G, Pottle B, Dod J. Asthma and the motorways - one District's experience. *Journal of Public Health Medicine* 1995;17:85-9.

45. Venn A, Lewis S, Cooper M, Hill J, Britton J. Increasing prevalence of wheeze and asthma in Nottingham primary schoolchildren 1988-1995. *European Respiratory Journal* 1998;11:1324-8.

46. Austin JB, Russell G. Wheeze, cough, atopy and indoor environment in the Scottish Highlands. *Archives of Disease in Childhood* 1997;76:22-6.

47. Venn A, Lewis S, Cooper M, Hill J, Britton J. Questionnaire study of effect of sex and age on the prevalence of wheeze and asthma in adolescence. *British Medical Journal* 1998;316:1945-6.

48. Kuehni CE, Davis A, Brooke AM, Silverman M. Are all wheezing disorders in very young (preschool) children increasing in prevalence? *Lancet* 2001;357:1821-5.

49. Shamssain MH, Shamsian N. Prevalence and severity of asthma, rhinitis and atopic eczema: the north east study. *Archives of Disease in Childhood* 1999;81:313-7.

50. Ng-Man-Kwong G, Das C, Proctor A, Whyte MKB, Primhak RA. Diagnostic and treatment behaviour in children with chronic respiratory symptoms: relationship with socioeconomic factors. *Thorax* 2002;57:701-4.

51. Kurukulaaratchy RJ, Fenn M, Twiselton R, Matthews S, Arshad SH. The prevalence of asthma and wheezing illnesses amongst 10-year-old schoolchildren. *Respiratory Medicine* 2002;96:163-9.

52. Frank PI, Wicks PD, Hazell ML, Linehan MF, Hirsch S, Hannaford PC, Frank TL. Temporal change in the prevalence of respiratory symptoms and obstructive airways disease 1993-2001. *British Journal of General Practice* 2005;55(517):596-602.

53. Martindale S, McNeill G, Devereux G, Campbell D, Russell G, Seaton A. Antioxidant intake in pregnancy in relation to wheeze and eczema in the first two years of life. *American Journal of Respiratory and Critical Care Medicine* 2005;171(2):121-8.

54. McCann D, McWhirter J, Coleman H, Devall I, M. C, Weare K, Warner J. The prevalence and management of asthma in primary-aged schoolchildren in the south of England. *Health Education Research* 2002;17:181-94.

55. Anderson HR, Ruggles R, Strachan DP, Austin JB, Burr M, Jeffs D, Standring P, Steriu A, Goulding R. Trends in prevalence of symptoms of asthma, hay fever, and eczema in 12-14 year olds in the British Isles, 1995-2002: questionnaire survey. *British Medical Journal* 2004;328:1052-3.

56. Lewis SA, Antoniak M, Venn AJ, Davies L, Goodwin A, Salfield N, Britton J, Fogarty AW. Secondhand smoke, dietary fruit intake, road traffic exposures, and the prevalence of asthma: a cross-sectional study in young children. *American Journal of Epidemiology* 2005;161(5):406-11.

57. ISAAC Steering Committee. Worldwide variation in prevalence symptoms of asthma, allergic rhinoconjunctivitis and atopic eczema: ISAAC. *Lancet* 1998;351:1225-32.
