# Supplementary material for: Systematic review of worldwide variations of the prevalence of wheezing symptoms in children
Source: Environ Health. 2008 Nov 10;7:57. doi: 10.1186/1476-069X-7-57 (PMC2614981; doi:10.1186/1476-069X-7-57)
Supplement: Additional file 3 — Studies of wheeze prevalence in the Eastern Mediterranean and Africa. As in Additional file 1. [file 1476-069X-7-57-S3.doc]

**Additional File 3. Studies of wheeze prevalence in the Eastern Mediterranean and Africa**

| **Country** | **Reference** | **Survey Year** | **Area** | **N (Response rate)** | **Age (years) / ascertainment**  (P=Parental-report  S=Self-report) | **Prevalence**  **%** | **95% CI** |
| --- | --- | --- | --- | --- | --- | --- | --- |
| **Algeria** | [1] | ’94-‘95 | Algiers | 1,147 (97.8%) | 13-14 S | 7.8 ^IS^ | 7.3, 8.3^*^ |
| **Ethiopia** | [1] | ’94-‘95 | Addis Ababa, Jima | 5,906 (98.8%) | 13-14 S | 6.2 ^IS^ | 5.6, 6.8^*^ |
|  | [2] | ’96 | Jimma and 3 rural communities | 2,929:  3,957: | 0-9: P  10-19: S | 2.0 ^D^  1.7 ^D^ | 1.5, 2.5^*^  1.3, 2.1^*^ |
|  | [3] | ’97 | Gondar Zuria, Dembia, Wegera, Lai Armachiho | 3,365 (98.4%) | 13-14 S | 16.2 ^IQ^ | 15.0, 17.4^*^ |
|  | [4] | ’00-‘01 | Jimma & 3 rural communities | 7,155 | 1-4 P | 3.4 ^IQ^ | 3.0, 3.8^*^ |
| **Iran** | [1] | ’94-‘95 | Rasht, Tehran | 5,469^†^ (>80%):  5,474 (93.2%): | 6-7: P  13-14: S | 5.4 ^IS^  10.9 ^IS^ | 4.8, 6.0^*^  10.1, 11.7^*^ |
| **Israel** | [5] | ’97 | National | 10,057 (86.4%): | 13-14: S | 17.9 ^IQ^ | 17.2, 18.6^*^ |
| **Kenya** | [6] | ’93 | Muranga, Nairobi | 1,172 (91.8%) | 10 P | 5.9 ^E^ | 4.6, 7.2^*^ |
|  | [1] | ’94-‘95 | Eldoret, Nairobi | 6,236 (99.5%) | 13-14 S | 13.9 ^IS^ | 13.0, 14.8^*^ |
|  | [7] | ’95:  ’01: | Uasin Gishu district | 3,018:  3,258: | 13-14: S  13-14: S | 10.2 ^IQ^  13.8 ^IQ^ | 9.1, 11.3^*^  12.6, 15.0^*^ |
| **Kuwait** | [8] | ’95 & ’96 | National | 3,110 | 13-14 S | 16.1 ^IS^ | 14.8, 17.4 |
| **Lebonan** | [1] | ’94-‘95 | Beirut | 2,993 (100%) | 13-14 S | 14.4 ^IS^ | 13.1, 15.7^*^ |
| **Malta** | [1] | ’94-‘95 | Nationwide | 3,493^†^ (>80%):  3,711 (88.7%): | 6-7: P  13-14: S | 8.8 ^IS^  16.0 ^IS^ | 7.9, 9.7^*^  14.9, 17.1^*^ |
| **Morocco** | [1] | ’94-‘95 | Casablanca, Marrakech, Rabat | 8,900 (95.1%) | 13-14 S | 7.5 ^IS^ | 7.8, 8.0^*^ |
| **Nigeria** | [1] | ’95 | Ibadan | 3,057 (76.5%) | 13-14 S | 10.7 ^IS^ | 9.6, 11.8^*^ |
| **Oman** | [1] | ’94-‘95 | Al-Khod | 3,891^†^ (>80%):  2,984 (94.0%): | 6-7: P  13-14: S | 7.1 ^IS^  8.9 ^IS^ | 6.3, 7.9^*^  7.9, 9.9^*^ |
| **Palestine**  **(West Bank)** | [9] | ’00 | Ramallah | 1,048 (86%)  2,334 (86%) | 6-7: P  8-12: P | 10.2 ^IQ^  8.4 ^IQ^ | 8.4, 12.0^*^  7.3, 9.5^*^ |
|  | [10] | ’00-‘01 | Palestine, Ramallah, North Gaza | 14,650 (84%):  14,060 (90%): | 5-8: P  12-15: S | 9.5 ^IQ^  7.3 ^IQ^ | 9.0, 10.0^*^  6.9, 7.7^*^ |
| **South Africa** | [11] | ‘90 | Cape Town | 1,239 (98.9%) | 12 P | 17.8 ^D^ | 15.7, 19.9^*^ |
|  | [12] | ’93 | Mitchell’s Plain area | 1,955 (90.0%) | 7-8 P | 26.8 ^D^ | 24.8, 28.8 |
|  | [1] | ’94-‘95 | Cape Town | 4,283 (82.8%) | 13-14 S | 16.1 ^IS^ | 15.1, 17.1^*^ |
|  | [13] | Published ‘02 | Cape Town | 4,706 (82.8%) | 13-14 S | 16.0 ^IQ^ | 15.0, 17.0^*^ |
| **Turkey** | [14] | ’92: | Ankara | 1,036: | 6-13 P | 11.9 ^D^ | 10.0, 14.0 |
|  | [15] | ’94 | Edirne | 5,412 (85.8%): | 7-12 P | 5.8 ^D^ | 5.2, 6.4 |
|  | [16] | ’95 | Istanbul | 2,216 (94.9% ) | 6-12 P | 8.2 ^IQ^ | 7.1, 9.3 |
|  | [17] | ’96 | Ankara | 2,784 (88.3%) | 7-14 P | 4.7 ^A^ | 3.9, 5.5 |
|  | [18] | ’96-‘97 | Istanbul | 2276 (87.5%) | 6-15 P | 7.2 ^D^ | 6.1, 8.3^*^ |
|  | [19] | ‘96 | Nationwide | 14,492 (93.6%):  17,873 (93.6%):  14,412 (93.6%): | 0-4: P  5-10: P  11-17: P | 5.1 ^A^  3.2 ^A^  1.8 ^A^ | 4.7, 5.5^*^  2.9, 3.5^*^  1.6, 2.0^*^ |
|  | [20] | ’99-‘00 | Ankara | 3,041 (88.7%) | 8-11 P | 11.5 ^IQ^ | 10.4, 12.6^*^ |
|  | [21] | ’00-‘01 | Afyon | 1,366 (94.5%) | 13-18 S | 12.2 ^F^ | 10.5, 13.9^*^ |

Key:

‘Prevalence’

IS: ISAAC study, with question “Have you had wheezing and whistling in the chest in the last 12 months?” (Yes/No)

IQ: ISAAC question, but not an ISAAC study

A: In the past 12 months has your child had a wheezing or asthma attack? (Yes/No)

B: Current wheezing without a diagnosis of asthma & Physician diagnosed asthma

C: In the last 12 months, has a wheeze (that is, a whistling noise, high or low pitched) ever been heard from your child’s chest?

D: Has your child (ever) wheezed in the past 12 months?

E: Wheeze in the previous year (interview questionnaire)

F: Have you had wheezing attacks in the past year?

G: Has your child had wheezing in the chest (but not from the throat or nose)

H: Wheezy or whistling sound in the chest when having a cold or occasionally apart from colds or for most days or nights, in the past 12 months

^*^ CI not given in the publication and calculated by author

† N is the number of questionnaires given out & response rate obtained from ISAAC study [1,22]

**References**

1. ISAAC Steering Committee. Worldwide variations in the prevalence of asthma symptoms: the International Study of Asthma and Allergies in Childhood (ISAAC). *European Respiratory Journal* 1998;12:315-35.

2. Yemaneberhan H, Bekele Z, Venn A, Lewis S, Parry E, Britton J. Prevalence of wheeze and asthma and relation to atopy in urban and rural Ethiopia. *Lancet* 1997;350:85-90.

3. Hailu S, Tessema T, Silverman M. Prevalence of symptoms of asthma and allergies in schoolchildren in Gondar town and its vicinity, Northwest Ethiopia. *Pediatric Pulmonology* 2003;35:427-32.

4. Dagoye D, Bekele Z, Woldemichael K, Nida H, Yimam M, Hall A, Venn AJ, Britton JR, Hubbard R, Lewis SA. Wheezing, allergy, and parasite infection in children in urban and rural Ethiopia. *American Journal of Respiratory and Critical Care Medicine* 2003;167:1369-73.

5. Shohat T, Golan G, Tamir R, Green MS, Livne I, Davidson Y, Harari G, Garty BZ. Prevalence of asthma in 13-14 yr-old schoolchildren across Israel. *European Respiratory Journal* 2000;15:725-9.

6. Odhiambo JA, Ng'ang'a LW, Mungai MW, Gicheha CM, Nyamwaya JK, Karimi F, Macklem PT, Becklake MR. Urban-rural differences in questionnaire-derived markers of asthma in Kenyan school children. *European Respiratory Journal* 1998;12:1105-12.

7. Esamai F, Ayaya S, Nyandiko W. Prevalence of asthma, allergic rhinitis and dermatitis in primary school children in Uasin Gishu district, Kenya. *East African Medical Journal* 2002;79:514-8.

8. Behbehani NA, Abal A, Syabbalo NC, Abd-Azeem A, Shareef E, Al-Momen J. Prevalence of asthma, allergic rhinitis, and eczema in 13-14-year-old children in Kuwait: an ISAAC study. *Annals of Allergy, Asthma and Immunology* 2000;85:58-63.

9. El-Sharif N, Abdeen Z, Qasrawi R, Moens G, Nemery B. Asthma prevalence in children living in villages, cities and refugee camps in Palestine. *European Respiratory Journal* 2002;19:1026-34.

10. El-Sharif NA, Nemery B, Barghuthy F, Mortaja S, Qasrawi R, Abdeen Z. Geographical variations of asthma and asthma symptoms among schoolchildren aged 5 to 8 years and 12-15 years in Palestine: the ISAAC. *Annals of Allergy, Asthma and Immunology* 2003;90:63-71.

11. Burr ML, Limb ES, Andrae S, Barry DMJ, Nagel F. Childhood asthma in four countries: a comparative survey. *International Journal of Epidemiology* 1994;23:341-7.

12. Ehrlich RI, Du-Toit D, Jordaan E, Volmink JA, Weinberg EG, Zwarenstein M. Prevalence and reliability of asthma symptoms in primary schoolchildren in Cape Town. *International Journal of Epidemiology* 1995;24:1138-45.

13. Poyser MA, Nelson H, Ehrlich RI, Bateman ED, Parnell S, Puternam A, et al. Socioeconomic deprivation and asthma prevalence and severity in young adolescents. *European Respiratory Journal* 2002;19:892-8.

14. Kalyoncu AF, Selcuk ZT, Karakoca Y, Emri AS, Coplu L, Sahin AA, Baris YI. Prevalence of childhood asthma and allergic diseases in Ankara, Turkey. *Allergy* 1994;49:485-8.

15. Selcuk ZT, Caglar T, Enunlu T, Topal T. The prevalence of allergic diseases in primary school children in Edirne, Turkey. *Clinical and Experimental Allergy* 1997;27:262-9.

16. Ones U, Sapan N, Somer A, Disci R, Salman N, Guler N, Yalcin I. Prevalence of childhood asthma in Istanbul, Turkey. *Allergy* 1997;52:570-5.

17. Saraclar Y, Sekerel BE, Kalayci O, Cetinkaya F, Adalioglu G, Tuncer A, Tezcan S. Prevalence of asthma symptoms in school children in Ankara, Turkey. *Respiratory Medicine* 1998;92:203-7.

18. Akcakaya N, Kulak K, Hassanzadeh A, Camcioglu Y, Cokugras H. Prevalence of bronchial asthma and allergic rhinitis in Istanbul school children. *European Journal of Epidemiolology* 2000;16:693-9.

19. Turktas I, Selcuk ZT, Kalyoncu AF. Prevalence of asthma-associated symptoms in Turkish children. *Turkish Journal of Pediatrics* 2001;43:1-11.

20. Saraclar Y, Kuyucu S, Tuncer A, Sekerel B, Sackesen C, Kocabas C. Prevalence of asthmatic phenotypes and bronchial hyperresponsiveness in Turkish schoolchildren: an ISAAC phase 2 study. *Annals of Allergy, Asthma and Immunology* 2003;91:477-84.

21. Unlu M, Orman A, N. D. The prevalence of asthma among secondary school students in Afyon, Turkey. *Asian Pacific Journal of Allergy Immunolology* 2002;20:1-6.

22. ISAAC Steering Committee. Worldwide variation in prevalence symptoms of asthma, allergic rhinoconjunctivitis and atopic eczema: ISAAC. *Lancet* 1998;351:1225-32.
