# Supplementary material for: Systematic review of worldwide variations of the prevalence of wheezing symptoms in children
Source: Environ Health. 2008 Nov 10;7:57. doi: 10.1186/1476-069X-7-57 (PMC2614981; doi:10.1186/1476-069X-7-57)
Supplement: Additional file 4 — Studies of wheeze prevalence in Asia. As in Additional file 1. [file 1476-069X-7-57-S4.doc]

**Additional File 4**. Studies of wheeze prevalence in Asia

| **Country** | **Reference** | **Survey Year** | **Area** | **N (Response rate)** | **Age (years) / ascertainment**  (P=Parental-report  S=Self-report) | **Prevalence**  **%** | **95% CI** |
| --- | --- | --- | --- | --- | --- | --- | --- |
| **China** | [1] | ’94-‘95 | Beijing, Chongqing, Guangzhou, Shanghai, Wulumuqi | 18,704 (98.4%) | 13-14 S | 4.2 ^IS^ | 3.9, 4.5^*^ |
|  | [2] | ’95-‘96 | Beijing:  Urumqi: | 7,668 (98.9%) | 6-7 P | 6.0 ^IS^  2.9 ^IS^ | 5.1, 6.9  2.3, 3.5 |
|  | [3] | ’97-‘98 | Hong Kong:  Guangzhou:  Beijing: | 10,902 (94%) | 9-11 P | 5.8 ^IQ^  3.4 ^IQ^  3.8 ^IQ^ | 5.0, 6.7  2.8, 4.1  3.3, 4.4 |
|  | [4] | ’99 | Tou-Cheng City | 8,754 (97.2%) | 13-15 S | 8.2 ^IQ^ | 7.6, 8.8^*^ |
|  | [5] | ‘01 | Wuhun | 4,185 | 14-15 S | 4.2 ^IQ^ | 3.6, 4.8^*^ |
| **Hong Kong** | [6] | ’92 | Hong Kong | 1,062 (89.2%) | 11-20 P | 3.7 ^D^ | 2.6, 4.8 |
|  | [1] | ’95:  ’94-‘95: | Hong Kong | 3,509 (97%):  4,526 (97%): | 6-7: P  13-14: S | 9.1 ^IS^  12.4 ^IS^ | 8.1, 10.1^*^  11.4, 13.4^*^ |
|  | [7] | ‘95 | Hong Kong | 2,292 (87%) | 8-12 P | 10.8 ^H^ | 9.5, 12.1^*^ |
|  | [8] | ’97-‘98 | Hong Kong | 3,110 (97%) | 10 P | 6.6 ^IQ^ | 5.7, 7.5^*^ |
|  | [9] | ‘01 | Hong Kong | 4,448 (95%) | 6-7 P | 9.5 ^IQ^ | 8.5, 10.3^*^ |
|  | [10] | ‘02 | Hong Kong | 3,321 (99%) | 13-14 S | 8.7 ^IS^ | 7.8 – 9.7 |
| **India** | [1] | ’94-‘95 | 11 cities | 31,697^†^(>80%):  35,461(95.4%): | 6-7: P  13-14: S | 5.6 ^IS^  6.0 ^IS^ | 5.3, 5.9^*^  5.8, 6.2^*^ |
| **Indonesia** | [1] | ’94-‘95 | Bandung | 1,390^†^ (>80%):  2,152 (95.7%): | 6-7: P  13-14: S | 4.1 ^IS^  2.1 ^IS^ | 3.1, 5.1^*^  1.5, 2.7^*^ |
| **Japan** | [1] | ’94-‘95 | Fukuoka | 2,900^†^ (>80%):  2,667 (94.2%): | 6-7: P  13-14: S | 17.3 ^IS^  13.4 ^IS^ | 15.9, 18.7^*^  12.1, 14.7^*^ |
|  | [11] | ’01 | Suita City | 5,614 (76%) | 12-15 P | 6.7  ^IQ^ | 6.0, 7.4^*^ |
| **Korea** | [12] | ’95 | Seoul and Provincial cities | 5,494 (92.5%):  10,015(97.3%): | 6-7: P  13-14: S | 13.6 ^IQ^  7.9 ^IQ^ | 12.7, 14.6  7.4, 8.5 |
|  | [13] | ’98 | Rural areas | 2,087:  2,137:  3,030:  (91.2% overall) | 7-9: P  10-12: P  13-15: P | 11.5 ^IQ^  11.2 ^IQ^  9.4 ^IQ^ | 10.1, 12.9^*^  9.9, 12.5^*^  8.4, 10.4^*^ |
|  | [14] | ‘00 | Seoul & Provincial cities | 15,894 (96.4%) | 12-15 S | 9.3 ^IQ^ | 8.9, 9.8 |
|  | [15] | Published ‘01 | Urban and rural areas | 2,055 | 7-16 P | 8.2 ^IQ^ | 7.0, 9.4 |
|  | [16] | Published ‘01 | Industrial factories area & less polluted area | 6,886 (91.7%) | 7-12 P | 10.3 ^IQ^ | 9.6, 11.0 |
|  | [17] | Published ‘02 | Urban and rural areas | 16,624 (86.6%) | 7-18 P<10/S | 9.3 ^IQ^ | 8.9, 9.7^*^ |
|  | [18] | Published‘02 | Rural areas | 1,727 (86.1%) | 16-18 S | 13.0 ^IQ^ | 11.4, 14.6^*^ |
| **Malaysia** | [1] | ’94-‘95 | Alor Setar, Ipoh, Klang Valley, Kota Bharu, Muar | 15,285^†^(>80%):  17,313(92.9%): | 6-7: P  13-14: S | 6.1 ^IS^  9.6 ^IS^ | 5.7, 6.5^*^  9.2, 10.0^*^ |
|  | [19] | ’01: | Kota Bharu | 3,157:  3,004: | 6-7: P  13-14: S | 4.3 ^IQ^  5.7 ^IQ^ | 3.6, 5.0^*^  4.9, 6.5^*^ |
| **Pakistan** | [1] | ’94-‘95 | Karachi | 1,829 (100%) | 13-14 S | 8.5 ^IS^ | 7.2, 9.8^*^ |
| **Philippines** | [1] | ’94-‘95 | Metro Manilla | 3,558^†^ (>80%):  3,063 (95.5%): | 6-7: P  13-14: S | 11.3 ^IS^  12.3 ^IS^ | 10.3, 12.3^*^  11.2, 13.4^*^ |
| **Singapore** | [1] | ’94 | Nationwide | 2,118 (90%):  3,785 (90%): | 6-7: P  13-14: S | 15.7 ^IS^  9.7 ^IS^ | 14.2, 17.2^*^  8.8, 10.6^*^ |
|  | [20] | ‘01 | Nationwide | 5,305:  4,058: | 6-7: P  12-15: S | 10.2 ^IQ^  11.9 ^IQ^ | 9.4, 11.0^*^  10.9, 12.9^*^ |
| **Taiwan** | [1] | ’94-‘95 | Taipei | 4,806^†^ (>80%):  10,636(93.3%): | 6-7: P  13-14: S | 9.6 ^IS^  5.2 ^IS^ | 8.8, 10.4^*^  4.8, 5.6^*^ |
|  | [21] | ‘02 | Taoyuan | 3,079:  3,111: | 6-8: P  13-15: S | 7.5 ^IQ^  4.2 ^IQ^ | 6.6, 8.5  3.5, 5.3 |
| **Thailand** | [1] | ’95 | Bankok | 3,629^†^ (>80%):  2,777 (74.8%): | 6-7: P  13-14: S | 11.0 ^IS^  13.5 ^IS^ | 10.0, 12.0^*^  12.2, 14.8^*^ |
|  | [1] | ’95 | Chiang Mai | 3,522 (92%):  3,731 (95%): | 6-7: P  13-14: S | 5.5 ^IS^  12.6 ^IS^ | 4.7, 6.3^*^  11.5, 13.7^*^ |

Key:

‘Prevalence’

IS: ISAAC study, with question “Have you had wheezing and whistling in the chest in the last 12 months?” (Yes/No)

IQ: ISAAC question, but not an ISAAC study

A: In the past 12 months has your child had a wheezing or asthma attack? (Yes/No)

B: Current wheezing without a diagnosis of asthma & Physician diagnosed asthma

C: In the last 12 months, has a wheeze (that is, a whistling noise, high or low pitched) ever been heard from your child’s chest?

D: Has your child (ever) wheezed in the past 12 months?

E: Wheeze in the previous year (interview questionnaire)

F: Have you had wheezing attacks in the past year?

G: Has your child had wheezing in the chest (but not from the throat or nose)

H: Wheezy or whistling sound in the chest when having a cold or occasionally apart from colds or for most days or nights, in the past 12 months

^*^ CI not given in the publication and calculated by author

† N is the number of questionnaires given out & response rate obtained from ISAAC study [1,22]

**References**

1. ISAAC Steering Committee. Worldwide variations in the prevalence of asthma symptoms: the International Study of Asthma and Allergies in Childhood (ISAAC). *European Respiratory Journal* 1998;12:315-35.

2. Zhao TB, Wang A, Chen Y, Xiao M, Duo L, Lui G, Lau YL, Karlberg J. Prevalence of childhood asthma, allergic rhinitis and eczema in Urumqi and Beijing. *Journal of Paediatrics and Child Health* 2000;36:128-33.

3. Wong GW, Hui DS, Chan HH, Fok TF, Leung R, Zhong NS, Chen YZ, Lai CKW. Prevalence of respiratory and atopic disorders in Chinese schoolchildren. *Clinical and Experimental Allergy* 2001;31:1225-31.

4. Chen CF, Wu KG, Hsu MC, Tang RB. Prevalence and relationship between allergic diseases and infectious diseases. *Journal of Microbiology, Immunology and Infection* 2001;34:57-62.

5. Salo PM, Xia J, Johnson CA, Li Y, Avol EL, Gong J, London SJ. Indoor allergens, asthma, and asthma-related symptoms among adolescents in Wuhan, China. *Annals of Epidemiology* 2004;14(8):543-50.

6. Leung R, Ho P. Asthma, allergy and atopy in three Southeast Asian populations. *Thorax* 1994;49:1205-10.

7. Yu ITS, Wong TW, Li W. Using child reported respiratory symptoms to diagnose asthma in the community. *Archives of Disease in Childhood* 2004;89(6):544-8.

8. Wong GW, Hui DS, Tam CM, Chan HH, Fok TF, Chan-Yeung M, Lai CKW. Asthma, atopy and tuberculin responses in Chinese schoolchildren in Hong Kong. *Thorax* 2001;56:770-3.

9. Lee S-L, Wong W, Lau Y-L. Increasing prevalence of allergic rhinitis but not asthma among children in Hong Kong from 1995 to 2001 (Phase 3 International Study of Asthma and Allergies in Childhood). *Pediatric Allergy and Immunology* 2004;15(1):72-8.

10. Wong GWK, Leung TF, Ko FWS, Lee KKM, Lam P, Hui DSC, FOK TF, Lai CKW. Declining asthma prevalence in Hong Kong Chinese schoolchildren. *Clinical And Experimental Allergy* 2004;34(10):1550-5.

11. Miyake Y, Yura A, Masayuki I. Relationship between distance from major roads and adolescent health in Japan. *Journal of Epidemiology* 2002;12:418-23.

12. Lee SI, Shin MH, Lee HB, Lee JS, Son BK, Koh YY, Kim KE, Ahn YO. Prevalences of symptoms of asthma and other allergic diseases in Korean children: a nationwide questionnaire survey. *Journal of Korean Medical Science* 2001;16:155-64.

13. Kim YK, Park HS, Kim HY, Jee YK, Son JW, Bae JM, Lee MH, Cho SH, Min KU, Kim YY. Citrus red mite (Panonychus citri) may be an important allergen in the development of asthma among exposed children. *Clinical and Experimental Allergy* 2001;31:582-9.

14. Hong S-J, Lee M-S, Sohn MH, Shim JY, Han YS, Park KS, Ahn YM, Son BK, Lee HB. Self-reported prevalence and risk factors of asthma among Korean adolescents: 5-year follow-up study, 1995-2000. *Clinical and Experimental Allergy* 2004;34(10):1556-62.

15. Lee MH, Kim YK, Min KU, Lee BJ, Bahn JW, Son JW, Cho SH, Park HS, Koh YY, Kim YY. Differences in sensitization rates to outdoor aeroallergens, especially citrus red mite (Panonychus citri), between urban and rural children. *Annals of Allergy, Asthma and Immunology* 2001;86:691-5.

16. Kim YK, Baek D, Koh YI, Cho SH, Choi IS, Min KU, Kim YY. Outdoor air pollutants derived from industrial processes may be causally related to the development of asthma in children. *Annals of Allergy, Asthma and Immunology* 2001;86:456-60.

17. Kim YK, Chang YS, Lee MH, Hong SC, Bae JM, Jee YK, Chun BR, Cho SH, Min KU, Kim YY. Role of environmental exposure to spider mites in the sensitization and the clinical manifestation of asthma and rhinitis in children and asolescents living in rural and urban areas. *Clinical and Experimental Allergy* 2002;32:1305-9.

18. Kim SH, Kim YK, Lee MH, Hong SC, Bae JM, Min KU, Kim YY, Cho SH. Relationship between sesitization to citrus red mite (Panonychus citri) and the prevalence of atopic diseases in adolescents living near citrus orchards. *Clinical and Experimental Allergy* 2002;32:1054-8.

19. Quah BS, Wan-Pauzi I, Ariffin N, Mazidah AR. Prevalence of asthma, eczema and allergic rhinitis: two surveys, 6 years apart, in Kota Bharu, Malaysia. *Respirology* 2005;10(2):244-9.

20. Wang XS, Tan TN, Shek LPC, Chng SY, Hia CPP, Ong NBH, Ma S, Lee BW, Goh DYT. The prevalence of asthma and allergies in Singapore; data from two ISAAC surveys seven years apart. *Archives of Disease in Childhood* 2004;89(5):423-6.

21. Kao C-C, Huang J-L, Ou L-S, See L-C. The prevalence, severity and seasonal variations of asthma, rhinitis and eczema in Taiwanese schoolchildren. *Pediatric Allergy and Immunology* 2005;16(5):408-15.

22. ISAAC Steering Committee. Worldwide variation in prevalence symptoms of asthma, allergic rhinoconjunctivitis and atopic eczema: ISAAC. *Lancet* 1998;351:1225-32.
