# Supplementary material for: Systematic review of worldwide variations of the prevalence of wheezing symptoms in children
Source: Environ Health. 2008 Nov 10;7:57. doi: 10.1186/1476-069X-7-57 (PMC2614981; doi:10.1186/1476-069X-7-57)
Supplement: Additional file 5 — Studies of wheeze prevalence in Australasia. As in Additional file 1. [file 1476-069X-7-57-S5.doc]

**Additional File 5. Studies of wheeze prevalence in Australasia**

| **Country** | **Reference** | **Survey Year** | **Area** | **N (Response rate)** | **Age (years) / ascertainment**  (P=Parental-report  S=Self-report) | **Prevalence**  **%** | **95% CI** |
| --- | --- | --- | --- | --- | --- | --- | --- |
| **Australia** | [1] | ’90: | Melbourne | 3,325 (89%):  2,899 (89%):  2,968 (89%): | 7: P  12: P  15: P | 23.1 ^A^  20.9 ^A^  18.6 ^A^ | 21.7, 24.5  19.4, 22.4  17.0, 19.8 |
|  | [2] | ’90 | Sidney, Melbourne, Brisbane, Hunter Valley | 8,753 (84%) | 8.7(Mean),  2.1(SD) P | 19.5 ^D^ | 18.7, 20.3^*^ |
|  | [3] | ’90-‘91 | Campbelltown | 4,550 (82%) | 12-18 S | 18.1 ^D^ | 17.0, 19.2^*^ |
|  | [4] | ’91 | Adelaide & Sydney | 2,947 (87%) | 12-15 S | 29.7 ^IQ^ | 28.1, 31.1^*^ |
|  | [5] | ‘92 | Burra, Gladstone, Kingston | 1,032 (80.0%) | 5-18 P | 25.0 ^D^ | 22.4, 27.6^*^ |
|  | [6] | ’93 | Sydney, Brisbane, Melborne, & Hunter Valley NSW | 10,106 (84.4%) | 5-12 P | 21.2 ^D^ | 20.4, 22.0^*^ |
|  | [7] | ’91-‘93 | State of NSW | 6,388 (78.4%) | 8-11 P | 24.1 ^D^ | 23.1, 25.1^*^ |
|  | [8] | ‘93 | South Australian state | 14,124 (72.8%) | 4 P | 25.2 ^D^ | 24.8, 25.6 |
|  | [9] | ’93 &’94 | Melbourne, Sydney, Adelaide, Perth | 9,155 (84%):  11,541 (94%): | 6-7: P  13-14: S | 24.6 ^IS^  29.4 ^IS^ | 23.7, 25.5^*^  28.6, 30.2^*^ |
|  | [10] | ’95 | Tazmania | 6,378 (92%) | 7 P | 22.0 ^IQ^ | 21.0, 23.0^*^ |
|  | [11] | ’97 | Moree, Wagga Wagga | 1,499 (67.3%) | 7-12 P | 27.7 ^D^ | 25.4, 30.0^*^ |
|  | [12] | Published ’99 | Melbourne, Government schools | 9,794 (75%) | 13-19 P  mainly 14-15 | 18.9 ^A^ | 18.0, 19.9 |
|  | [13] | ‘99 | Torres Strait & Northern Peninsula area | 1,650 (98%) | 0-17 P | 12.4 ^IQ^ | 10.8, 14.0 |
|  | [14] | ’99-‘01 | Aboriginal & Torres Strait Islander and Australian Capital Territory | 10,452 (80%) | 4-6 P | 15.5 ^IQ^ | 14.8, 16.2^*^ |
| **Fiji** | [15] | ’90 Sept | Suva city | 2,117 (97.4%) | 9-10 P | 21.0 ^IQ^ | 19.2, 22.8 |
| **New Zealand** | [16] | ’85:  ’91: | Auckland:  Auckland: | 1,084:  1,901 (80.4%): | 7-10: P  7-10: P | 14.8 ^D^  18.7 ^D^ | 12.7, 16.9  16.9, 20.5 |
|  | [4] | ’91 | Wellington | 1,863 (87%) | 12-15 S | 28.3 ^IS^ | 26.3, 30.3^*^ |
|  | [9] | ’92-‘93 | Auckland, Bay of Plenty, Christchurch, Hawke’s Bay, Nelson, Wellington | 16,898 (91%):  19,023 (93%): | 6-7: P  13-14: S | 24.5 ^IS^  30.2 ^IS^ | 23.9, 25.1^*^  29.5, 30.9^*^ |
|  | [17] | ‘00 | City of Hastings & Borough of Havelock North | 1,287 (84%) | 10-12 P | 22.0 ^IQ^ | 19.7, 24.3^*^ |
|  | [18] | ‘02 | Wellington | 2,539 (47%) | 6-7 P | 24.3 ^IQ^ | 22.6, 26.0^*^ |

Key:

‘Prevalence’

IS: ISAAC study, with question “Have you had wheezing and whistling in the chest in the last 12 months?” (Yes/No)

IQ: ISAAC question, but not an ISAAC study

A: In the past 12 months has your child had a wheezing or asthma attack? (Yes/No)

B: Current wheezing without a diagnosis of asthma & Physician diagnosed asthma

C: In the last 12 months, has a wheeze (that is, a whistling noise, high or low pitched) ever been heard from your child’s chest?

D: Has your child (ever) wheezed in the past 12 months?

E: Wheeze in the previous year (interview questionnaire)

F: Have you had wheezing attacks in the past year?

G: Has your child had wheezing in the chest (but not from the throat or nose)

H: Wheezy or whistling sound in the chest when having a cold or occasionally apart from colds or for most days or nights, in the past 12 months

^*^ CI not given in the publication and calculated by author

† N is the number of questionnaires given out & response rate obtained from ISAAC study [9,19]

**References**

1. Robertson CF, Bishop J, Sennhauser FH, Mallol J. International comparison of asthma prevalence in children: Australia, Switzerland, Chile. *Pediatric Pulmonology* 1993;16:219-26.

2. Bauman A, Mitchell CA, Henry RL, Robertson CF, Abramson MJ, Comino EJ, Hensley MJ, Leeder SR. Asthma morbidity in Australia: an epidemiological study. *Medical Journal of Australia* 1992;156:827-31.

3. Forero R, Bauman A, Young L, Larkin P. Asthma prevalence and management in Australian adolescents: results from three community surveys. *Journal of Adolescent Health* 1992;13:707-12.

4. Pearce NE, Weiland S, Keil U, Langridge P, Anderson HR, Strachan D, Bauman A, Young L, Gluyas P, Ruffin D, Crane J, Beasley R. Self-reported prevalence of asthma symptoms in children in Australia, England, Germany and New Zealand: An international comparison using the ISAAC written and video questionnaires. *European Respiratory Journal* 1993;6:1455-61.

5. Crockett AJ, Cranston JM, Alpers JH. The changing prevalence of asthma-like respiratory symptoms in South Australian rural schoolchildren. *Journal of Paediatrics and Child Health* 1995;31:213-7.

6. Comino EJ, Mitchell CA, Bauman A, Henry RL, Robertson CF, Abramson MJ, Ruffin R, Landau L. Asthma management in eastern Australia, 1990 and 1993. *Medical Journal of Australia* 1996;164:403-6.

7. Peat JK, Toelle BG, Gray EJ, Haby MM, Belousova E, Mellis CM, Woolcock AJ. Prevalence and severity of childhood asthma and allergic sensitisation in seven climatic regions of New South Wales. *Medical Journal of Australia* 1995;163:22-6.

8. Volkmer RE, Ruffin RE, Wigg NR, Davies N. The prevalence of respiratory symptoms in South Australian preschool children: I. Geographic location. *Journal of Paediatrics and Child Health* 1995;31:112-5.

9. ISAAC Steering Committee. Worldwide variations in the prevalence of asthma symptoms: the International Study of Asthma and Allergies in Childhood (ISAAC). *European Respiratory Journal* 1998;12:315-35.

10. Ponsonby AL, Couper D, Dwyer T, Carmichael A. Cross sectional study of the relation between sibling number and asthma, hay fever, and eczema. *Archives of Disease in Childhood* 1998;79:328-33.

11. Downs SH, Marks GB, Belosouva EG, Peat JK. Asthma and hayfever in Aboriginal and non-Aboriginal children living in non-remote rural towns. *Medical Journal of Australia* 2001;175:10-3.

12. Powell CV, Nolan TM, Carlin JB, Bennett CM, Johnson PD. Respiratory symptoms and duration of residence in immigrant teenagers living in Melbourne, Australia. *Archives of Disease in Childhood* 1999;81:159-62.

13. Valery PC, Chang AB, Shibasaki S, Gibson O, Purdie DM, Shannon C, Masters IB. High prevalence of asthma in five remote indigenous communities in Australia. *European Respiratory Journal* 2001;17:1089-96.

14. Glasgow NJ, Goodchild EA, Yates R, Ponsonby AL. Respiratory health in Aboriginal and Torres Strait Islander children in the Australian Capital Territory. *Journal of Paediatrics and Child Health* 2003;39:534-9.

15. Flynn MGL. Respiratory symptoms, bronchial responsiveness, and atopy in Fijian and Indian children. *American Journal of Respiratory and Critical Care Medicine* 1994;150:415-20.

16. Mitchell EA, Asher MI. Prevalence, severity and medical management of asthma in European schoolchildren in 1985 and 1991. *Journal of Paediatrics and Child Health* 1994;30:398-402.

17. Wickens K, Barry D, Friezema A, Rhodius R, Bone N, Purdie G, Crane J. Obesity and asthma in 11-12 year old New Zealand children in 1989 and 2000. *Thorax* 2005;60(1):7-12.

18. Cohet C, Cheng S, MacDonald C, Baker M, Foliaki S, Huntington N, Douwes J, Pearce N. Infections, medication use, and the prevalence of symptoms of asthma, rhinitis, and eczema in childhood. *Journal of Epidemiology and Community Health* 2004;58(10):852-7.

19. ISAAC Steering Committee. Worldwide variation in prevalence symptoms of asthma, allergic rhinoconjunctivitis and atopic eczema: ISAAC. *Lancet* 1998;351:1225-32.
